# Investigation of multi-performance optimization of banana/bark cloth reinforced epoxy composites using grey relational analysis for automotive interior applications

**DOI:** 10.1038/s41598-026-45783-9

**Published:** 2026-03-23

**Authors:** Alex Turyahabwe, Milon Selvam Dennison, Onep Samuel George

**Affiliations:** Department of Mechanical Engineering, School of Engineering and Applied Sciences, Kampala International University, Western Campus, Ishaka-Bushenyi, Uganda

**Keywords:** Natural fiber composites, Grey Relational Analysis, Banana fiber, Bark cloth, Mechanical properties, Flame retardancy, Epoxy composites, Automotive interiors, Engineering, Materials science

## Abstract

This study presents a novel multi-performance optimization of underexplored hybrid banana- and bark-cloth fiber-reinforced epoxy composites for automotive interior applications, using Grey Relational Analysis (GRA). Unlike previous studies that mainly examine banana fiber alone or other conventional natural fiber hybrids, the present work investigates the synergistic combination of banana fiber and bark cloth in an epoxy matrix with 5 wt% aluminium trihydrate (ATH) to simultaneously improve mechanical performance, flame resistance, and moisture-related durability. The composites were produced through compression moulding with varying fiber ratios, and their tensile, flexural, impact, flammability, and water absorption behaviours were systematically evaluated. Among the tested configurations, Sample C7 (40% banana, 5% bark cloth) exhibited the best overall performance, with tensile strength of 28.29 MPa, flexural strength of 37.86 MPa, specific impact energy of 106.92 J/g, and a burning rate of 1.053 mm/s. GRA was used to rank the composite formulations based on multiple response criteria, identifying C7 as the optimal configuration. SEM, EDAX, and FTIR analyses further confirmed improved fibre-matrix interaction, additive retention, and chemical compatibility. The findings demonstrate the potential of banana/bark cloth hybrid composites as sustainable multifunctional materials for automotive interiors.

**Supplementary Information:**

The online version contains supplementary material available at 10.1038/s41598-026-45783-9.

## Introduction

The global push towards sustainable Natural Fiber Composites (NFCs) for automotive interior applications is driven by the need to reduce environmental impact, enhance fuel efficiency through weight reduction, and thus address the depletion of petroleum resources^1,2^. NFCs offer a promising alternative to traditional synthetic materials due to their biodegradability, low manufacturing costs, and satisfactory mechanical properties, making them suitable for various automotive components, such as door panels, seatbacks, and trunk linings^3–5^. The automotive industry is increasingly adopting NFCs, leveraging fibers such as flax, jute, and hemp, which offer competitive mechanical properties and are more environmentally friendly than synthetic fibers^6,7^. Recent advancements in NFCs include the development of high-performance composites through techniques such as fiber hybridization, surface fibrillation, and the incorporation of graphene materials to enhance mechanical and interfacial properties^8–10^. Despite these advancements, challenges such as fiber-matrix compatibility, thermal stability, and water absorption persist, necessitating ongoing research to optimize processing techniques and improve the durability of NFCs^11–13^. The change towards NFCs in the automotive sector is not only a response to environmental regulations but also a strategic move to achieve cost-effectiveness and sustainability in vehicle manufacturing^14^.

The integration of NFCs with both mechanical robustness, resistance to water absorption, and flame-retardant capabilities has emerged as a pivotal concern in the design of automotive interior components^15,16^. The automotive industry’s shift toward sustainability is increasingly coupled with regulatory demands for fire-safe materials, especially in enclosed vehicle environments where passenger safety is paramount^16^. However, the use of natural fibers, while environmentally advantageous, presents challenges in achieving balanced performance across structural and flammability domains^17,18^. Recent advances in NFCs highlight their potential to replace petroleum-based reinforcements, offering advantages like biodegradability, lower density, and cost-effectiveness^19–21^. However, studies demonstrate that the fire-retardant behavior of these materials is inconsistent and highly dependent on fiber composition, surface treatment, and matrix interaction. For example, a study on hemp/wool fiber composites found significant variation in limiting oxygen index (LOI) and UL-94 classifications based on fiber layering and hybridization strategies. Despite some configurations reaching acceptable LOI values (e.g., 21.45%), many NFCs still fall short of the stringent flammability standards required for automotive applications^22^. Moreover, most fire-retardant strategies in polymer composites rely on halogen-free additives and synergists such as aluminium hypophosphite and zinc borate, which are effective in synthetic matrices like PA66 but have not been systematically applied to natural fiber/epoxy systems. In one such recent study, these synergists improved both char formation and smoke suppression, suggesting the need for cross-application of flame-retardant systems to bio-based composites^23^.

The mechanical properties of NFCs can be further enhanced through hybridization with other natural/synthetic fibers, such as glass and carbon, which improves their performance in structural applications^24,25^. Additionally, the use of innovative resins, such as furan, which is derived from renewable resources, enhances the fire-resistant properties of NFCs, making them comparable to traditional phenolic resins and superior to polyester and epoxy systems^26^. Moreover, advancements in processing techniques, such as the use of woven-nonwoven textile preforms and surface fibrillation, have been demonstrated to enhance the mechanical and thermal properties of these composites, thereby improving their applicability in the automotive sector^8,27^. Hybrid NFCs are increasingly being explored for automotive interior applications due to their potential to reduce weight and enhance environmental sustainability. These composites combine natural fibers, such as coir, jute, and flax, with synthetic fibers like glass or carbon, resulting in materials that offer a balance of mechanical strength, cost-effectiveness, and reduced environmental impact. For example, coir and glass fiber-reinforced polypropylene composites have demonstrated significant improvements in tensile, flexural, and impact properties, while also being lighter and less water-absorbent compared to composites reinforced solely with coir fibers^28^. The integration of natural fibers with synthetic ones in hybrid composites not only enhances mechanical and thermal properties but also provides a synergistic effect that cannot be achieved with single fiber-reinforced materials^25,29^. Recent studies show that sustainable composite research is increasingly focused on lightweight, high-performance materials for transport and structural applications, while also emphasizing durability, multiscale understanding, and predictive design tools. These developments highlight the broader importance of optimizing natural fibre hybrid composites and support the relevance of the present banana/bark cloth epoxy system for automotive interior applications^30–33^.

NFCs are being utilized in various automotive components, including interior panels, due to their lightweight nature and high strength-to-weight ratio^34^. Additionally, the use of natural fibers, such as cellulose, in combination with inorganic fibers in polypropylene matrices has shown promise in reducing reliance on non-biodegradable materials while maintaining desirable mechanical properties for automotive applications^35^. The development of hybrid composites also involves optimizing fiber orientations and lamination sequences to meet specific design requirements, as seen in the design of composite automotive drive shafts^36^. Moreover, incorporating natural fibers into hybrid fleeces for automotive interiors can improve acoustical properties and enhance recycling capabilities, offering both technical and economic advantages^37^. The integration of banana fibers with various matrices, including epoxy, polyester, and polylactic acid (PLA), has been extensively studied to enhance tensile and flexural strengths, as well as improve water resistance. For example, the use of PLA coating and alkali treatment on Careya arborea and banana fiber epoxy composites significantly enhanced tensile and flexural strengths by 20.56% and 16.7%, respectively, while reducing water absorption by 47.6%^38^. Similarly, banana fiber nonwoven composites reinforced with epoxy and polyester matrices demonstrated improved tensile and flexural strengths, with alkali treatment and water repellent applications further enhancing these properties^39^. The incorporation of banana fibers into recycled high-density polyethylene (HDPE) composites also showed that fiber content significantly influences tensile strength, suggesting potential applications in construction materials^40^. Moreover, the development of PLA/banana fiber biocomposites using different molding techniques revealed significant improvements in mechanical and dynamic properties, with extrusion injection molding yielding the best results^41^. In the context of bark cloth fibers, studies have shown that bark cloth laminar epoxy composites exhibit considerable tensile and flexural strengths, making them suitable for applications such as automotive interior panels^42^. Additionally, the integration of banana fibers into recycled PET composites has been explored, demonstrating high stiffness and interfacial cohesion, which are crucial for sustainable material development^43^. Recent automotive-composite studies have similarly used systematic decision frameworks ranging from MCDM (e.g., VIKOR with sensitivity analysis) to machine-learning-based prediction of impact/cost trade-offs to screen sustainable laminate options, reinforcing the need for structured multi-response ranking methods such as GRA in material selection^44,45^.

**Table 1:** Recent GRA-based optimization studies in natural and hybrid fibre composites.

Literature	Fibre(s)/Composite System	Matrix/Composite Type	Targeted Performance Metrics Optimised via GRA
Ganesan et al.^46^,	Calotropis gigantea and Prosopis juliflora (hybrid natural fibres)	Epoxy-based hybrid composite	Multi-response mechanical optimisation (tensile, flexural, impact strength) using Taguchi-GRA
Esangbedo & Samuel^47^	Sisal and glass fibres (natural/synthetic hybrid)	Fibre-reinforced polymer composite (for aircraft applications)	Multi-objective optimisation (weight reduction, structural reliability) using Taguchi-GRA + Genetic Algorithm
Jamshaid et al.^48^,	Protex, Nomex, FR-Viscose, Pyron/Carbon (inherently fire-resistant blend)	Knitted fire-resistant fabric structures	Multi-response (flammability, comfort, mechanical serviceability) using Taguchi-GRA
Pichumani et al.^49^,	Banana fibre + multi-walled carbon nanotubes (MWCNTs)	Hybrid composite (Epoxy, Vinylester, GP resins)	Multi-response: mechanical (tensile, impact) and vibration properties using Taguchi-GRA
Kirubakaran et al.^50^,	Banana, coir, rice husk, sugarcane, wood (natural fillers) + hBN	Multiphase epoxy composite (natural filler + hBN)	Multi-response: thermal conductivity, electrical resistance via Grey Relational Grade Analysis (GRGA)
Balasubramanian et al.^51^,	Coconut Shell Powder (CSP, 5–25 wt%) + Silver Nanoparticles (AgNPs, 1–5 mM)	PVA-based hybrid biocomposite film	Taguchi-based GRA used for multi-response optimisation of mechanical (tensile strength, Young’s modulus, elongation) and thermal stability
Kirubakaran et al.^52^,	Coir fibre + hBN (1–5 wt%)	Epoxy hybrid composite (hand lay-up)	Deng’s Grey Relational method applied for multi-response optimisation (water absorption, corrosion rate)
Rasu et al.^53^,	Banana + Sisal fibres with Red Mud filler	Epoxy-based hybrid composite (compression moulded)	Multi-response mechanical optimisation: tensile, flexural, impact, shear strength, hardness
Gangil et al.^54^,	Banana fibre (NaHCO_3_ treated)	Polypropylene composite (compression moulded)	Multi-response optimisation of mechanical (tensile, flexural, impact) and water absorption using Taguchi–GRA

The summary of recent GRA‑based optimization studies on natural and hybrid fibre composites is presented in Table 1. In the case of banana and bark cloth fibers, banana has been widely studied for its potential as a mechanical reinforcement and for its eco-friendliness. In contrast, research on bark cloth remains limited. However, a critical gap exists in evaluating their combined performance when hybridized in an epoxy matrix, particularly using multi-criteria evaluation frameworks such as Grey Relational Analysis (GRA).

The motivation for selecting bark cloth over more widely studied fibers like jute or kenaf stems lies in its distinctive structural and functional characteristics. Bark cloth, derived from the inner bark of the fig tree (*Ficus natalensis*), is a naturally laminated, nonwoven material with high lignin content and a crimped texture, which enhances its ability to dissipate impact energy and withstand thermal degradation. These attributes make it particularly suited for applications such as automotive interiors, where mechanical integrity and flame resistance are critical. Moreover, its underexploited potential in composite applications, especially in synergy with high-strength fibers like banana, offers a novel direction for sustainable material development. This hybridization seeks to balance stiffness and impact absorption, a combination less attainable with conventional fibers like jute or kenaf. In addition, flame-retardant data for bark cloth composites are scarce, despite their potential for thermal insulation. Current literature lacks studies that simultaneously optimize mechanical and flammability performance, nor do they employ systematic tools to rank composite configurations based on multiple performance outcomes. Therefore, this research addresses a significant gap by proposing a multi-performance optimization of banana/bark cloth-reinforced epoxy composites using Grey Relational Analysis (GRA), targeting their application in automotive interiors, where both mechanical integrity and fire safety are non-negotiable. This integrated approach is crucial to bridge the divide between environmental sustainability and regulatory compliance in the next generation of automotive materials.

This research aims to fabricate hybrid banana/bark fiber-reinforced epoxy composites tailored for automotive interior applications and to evaluate their mechanical properties, including tensile, flexural, impact, flame-retardant behavior, and water absorption resistance. A central goal is to employ GRA to simultaneously rank and optimize the composite configurations across these performance metrics. It further seeks to understand how variations in fiber proportions and laminate configuration influence composite durability and fire resistance. The research is guided by critical questions, including how banana and bark cloth fibers interact within an epoxy matrix, the impact of hybridization on water absorption and flame performance, whether GRA can effectively optimize multifunctional properties, and which configurations yield the best balance of mechanical integrity, fire safety, and moisture resistance. The study is scoped to banana and bark cloth reinforcements in epoxy matrices, focusing specifically on automotive interior parts such as door panels and seatbacks, and involves performance evaluation through standardized tests, including ASTM D635 HB and water absorption assessment. However, it is limited to laboratory-scale fabrication and does not cover industrial scalability, aging effects, or the use of other polymer matrices.

## Materials and methods

### Materials

#### Banana fiber

Banana fibers were extracted from the pseudostem sheaths of banana plants collected in Ishaka–Bushenyi, Western Uganda (0°32′42″S, 30°08′18″E). These lignocellulosic fibers are widely recognized for their low density, moderate tensile strength, and renewability, making them suitable for natural fiber-reinforced composite applications^55^. The banana fibers were manually extracted from the pseudostem sheaths by mechanical scraping and retting to remove non-fibrous tissues, followed by thorough washing and sun-drying prior to alkali treatment. To enhance fiber–matrix interfacial adhesion, the fibers were treated with a 5 wt% sodium hydroxide (NaOH) solution to remove surface impurities, lignin, and hemicellulose. The NaOH and double-distilled water used for both the preparation of the alkali solution and the rinsing process were procured from Zicopp Uganda Limited, Kampala, Central Region, Uganda. Following alkali treatment, the fibers were thoroughly rinsed with double-distilled water until the pH reached neutrality to eliminate residual chemicals, then oven-dried at 60 °C for 24 h to remove moisture. Subsequently, the dried banana fibers were cut to an average length of 350 mm to promote consistent load transfer and improve dimensional consistency across composite samples. The density of banana fiber was adopted from validated literature as 1.35 g/cm^3^^56^ and is reported here as a reference material property. The final composite formulations in this study were established and reported on a weight-percentage (wt%) basis rather than through direct volume-fraction calculations.

#### Bark cloth

Bark cloth, obtained from *Ficus natalensis* trees, was sourced from a local craft shop in Owino Market, Kampala, Central Region, Uganda (0°18′49″N, 32°34′13″E). This naturally non-woven, manually processed material is known for its porosity, thermal insulation, and moderate stiffness, making it a promising reinforcement for hybrid composites. Prior to fabrication, bark cloth sheets were manually cleaned to remove surface impurities, cut to (350 × 135) mm to fit the mould cavity, and oven-dried at 60 °C for 24 h under the same conditions as banana fibers to minimize residual moisture. This step ensured consistent processing conditions and improved fibre–matrix interfacial bonding during curing. Bark cloth was used in its natural form without chemical modification to preserve its inherent flame-retardant and acoustic properties, which complement the structured morphology of banana fibers. The average density of bark cloth was taken as 0.33 g/cm^3^ based on literature data^57^, and is reported here as a reference material property. The final composite formulations in this study were established and reported on a weight-percentage (wt%) basis.

#### Epoxy resin and hardener

The matrix was composed of Lapox B-47 epoxy resin and Aradur 3486 polyamine hardener, both obtained from Zicopp Uganda Limited, Kampala, Central Region, Uganda. Lapox B-47 is recognized for its medium viscosity, strong adhesion, and thermal and chemical resistance, making it an ideal matrix for natural fiber composites. Aradur 3486 provides efficient room-temperature curing and forms a tough, cross-linked thermoset network when combined with Lapox B-47. This resin-hardener combination is commonly used in fiber-reinforced polymer systems for load-bearing applications^58^.

#### Aluminium trihydrate (ATH)

Aluminium Trihydrate (ATH) was incorporated at 5 wt% of the epoxy matrix to impart flame-retardant properties. ATH acts as a heat sink and smoke suppressant, decomposing to release water vapor during combustion, which in turn reduces surface temperature and dilutes combustible gases^59,60^. The ATH loading was fixed at 5 wt% based on prior reports^59,61^ indicating that this concentration provides an optimal balance between flame retardancy and mechanical performance without affecting resin viscosity or fiber wetting. In addition to the literature basis, ATH was kept at 5 wt% in this study to maintain consistent matrix processability across all laminates produced by hand lay-up followed by compression moulding. During initial mixing and lay-up handling, higher ATH loadings noticeably increased resin thickness and reduced ease of impregnation, which can promote non-uniform wet-out and void formation. To avoid confounding the influence of fibre hybridization with variability introduced by changes in resin flow and wetting behaviour, a single ATH level (5 wt%) was therefore adopted as a controlled baseline for all compositions. This additive was also sourced from Zicopp Uganda Limited, Kampala, Central Region, Uganda, ensuring high purity, halogen-free flame resistance, and mechanical performance without compromise.

### Composite fabrication

**Fig. 1:**
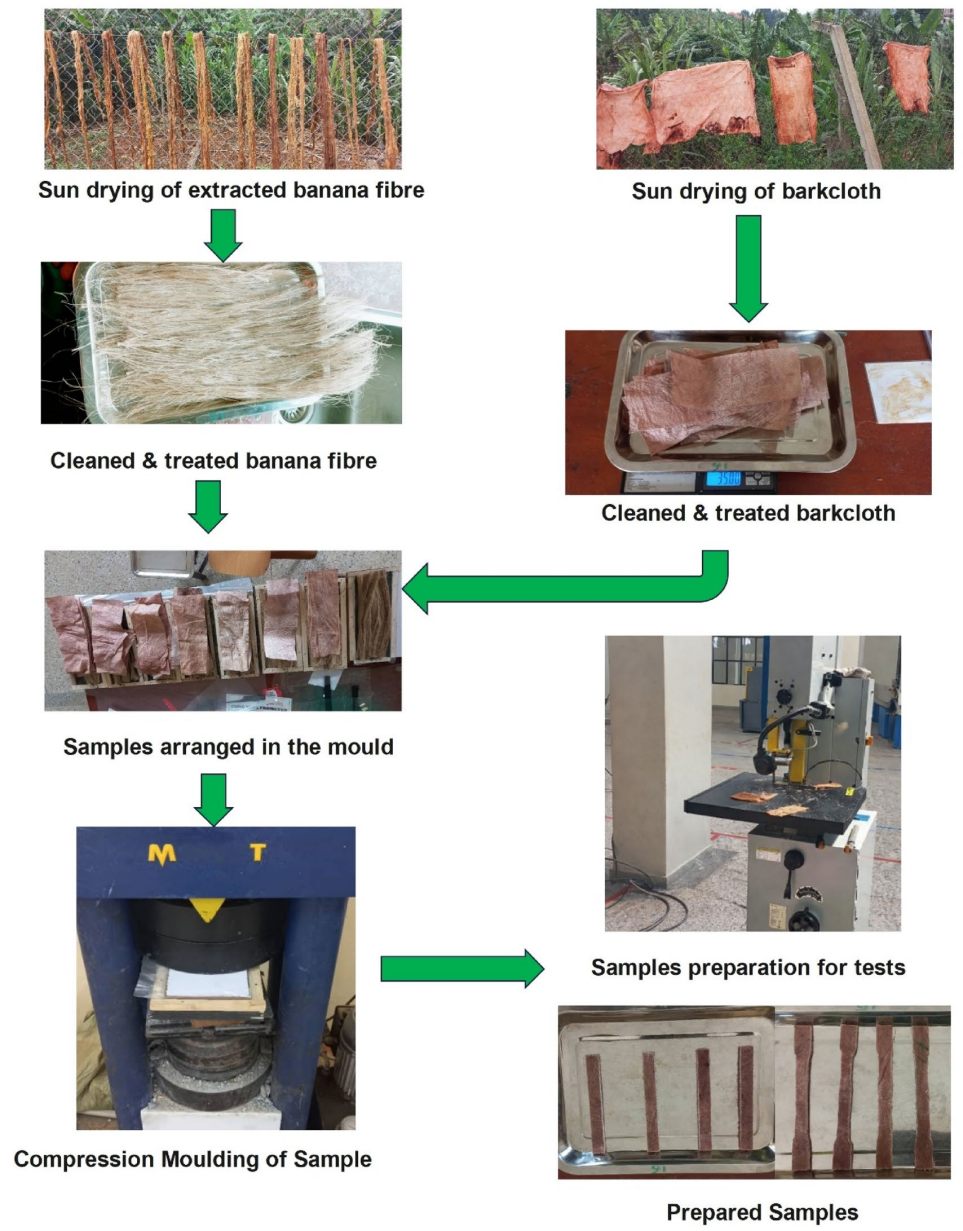
Illustration of sample preparation.

The hybrid composites were fabricated using the compression moulding technique, which applies uniform heat and pressure to enhance resin infiltration, reduce voids, and ensure effective fibre–matrix adhesion. This process enables better interfacial contact and structural consolidation, leading to improved mechanical consistency in the cured laminates. The illustration of sample preparation is depicted in Fig. 1.

#### Mould design and preparation

A custom-built wooden mould, internally lined with mica sheets, was used to fabricate composite laminates. Mica was selected for its thermal resistance, dimensional stability, and non-adherent surface properties, which help withstand moderate curing temperatures while facilitating smooth release of the composite laminate. The mould cavity was machined to produce plates with a nominal thickness of 3 mm and an effective surface area of (350 × 135) mm. Thus, laminate thickness was controlled primarily by the fixed mould geometry during compression moulding rather than by imposing an identical fibre volume fraction in all formulations. Prior to each lay-up, the mica-coated surfaces were cleaned with ethanol and coated with a thin film of silicone-based release agent to prevent matrix sticking and ensure a consistent surface finish.

#### Matrix formulation

The epoxy matrix consisted of Lapox B-47 resin and Aradur 3486 hardener mixed in a 100:40 weight ratio, as recommended by the manufacturer. To impart flame-retardant properties, 5 wt% aluminium trihydrate (ATH), a halogen-free flame retardant, was gradually incorporated into the resin mixture and stirred for 5 min to achieve uniform dispersion. Although the addition of ATH slightly increased the resin viscosity, it remained within a workable range for manual lay-up. Continuous mechanical stirring and degassing promoted homogeneous filler distribution and effective fibre wetting during processing. Although the void fraction was not quantitatively measured, all moulded composites were visually examined to confirm a smooth surface finish, and no obvious air entrapment was observed on the laminate surfaces after curing. The ATH content was limited to 5 wt% based on literature reports and preliminary trials, which indicated that higher loadings significantly increase resin viscosity and hinder fibre impregnation during hand lay-up. Maintaining this concentration ensured satisfactory flow behaviour and uniform filler dispersion, consistent with findings from previous studies^61,62^.

#### Lay-Up and compression moulding procedure

The fabrication of the hybrid banana/bark cloth epoxy composites was performed using a manual hand lay-up technique followed by compression moulding. The required quantities of fibres were weighed to match the desired weight fractions (wt%) and then uniformly distributed within the mould cavity. Each layer of banana fibre and bark cloth was carefully impregnated with the resin mixture using a roller and brush to ensure thorough wet-out and to eliminate entrapped air. The stacking sequence and number of layers were controlled to allow consolidation within the fixed 3 mm mould cavity for all composite configurations. Because the laminates contained different banana/bark cloth ratios and the two fibres have different densities and packing behaviour, the final 3 mm thickness should be interpreted as a nominal mould-controlled thickness rather than proof of identical fibre volume fraction or porosity across all samples. Each composite configuration (C1–C7) consisted of five layers arranged in accordance with the designed proportions of banana (B) and bark cloth (BC) fibers. A symmetric stacking sequence was used for hybrid laminates to maintain uniform mechanical response. The stacking details are presented in Table 2. It should be noted that the composite configurations differ not only in banana/bark cloth weight fraction but also, for some samples, in stacking arrangement, as shown in Table 2. Therefore, the mechanical and flammability responses obtained in this study reflect the combined influence of fibre composition and laminate architecture. Therefore, the mechanical and flammability responses reported in this study reflect the combined influence of fibre composition and laminate architecture, and the effect of stacking sequence cannot be separated from fibre composition in the present experimental design.

**Table 2:** Stacking sequence and number of layers for the fabricated banana/bark cloth epoxy composites.

Composite Sample	Stacking Sequence	Fiber Composition (wt%)	Remarks
C1	BC/B/BC/B/BC	10% Banana/35% Bark cloth	Bark cloth-dominant hybrid
C2	BC/B/BC/B/BC	15% Banana/30% Bark cloth	Bark cloth-rich hybrid
C3	B/BC/B/BC/B	20% Banana/25% Bark cloth	Balanced hybrid laminate
C4	B/BC/B/BC/B	25% Banana/20% Bark cloth	Near-balanced hybrid
C5	B/B/BC/B/B	30% Banana/15% Bark cloth	Banana-dominant hybrid
C6	B/B/BC/B/B	35% Banana/10% Bark cloth	Banana-rich hybrid
C7	B/BC/B/BC/B	40% Banana/5% Bark cloth	Banana-rich hybrid
C8	B/B/B/B/B	45% Banana/0% Bark cloth	Single-fiber composite (banana)

After lay-up, the mould was closed and placed under a 204-ton capacity hydraulic compression moulding machine (Matest S.p.A., Treviolo, Italy). A uniform compressive load of 20 kN was applied and maintained for 12 h at room temperature (≈ 25 °C) to allow gradual resin flow and curing, which facilitated good fibre wetting and consolidation without inducing thermal degradation of the natural fibres. Following demoulding, all samples underwent post-curing at 80 °C for 2 h in a convection oven to enhance cross-linking density, improve dimensional stability, and ensure complete polymerisation of the epoxy matrix. This combined ambient-pressure curing and controlled post-curing sequence provided excellent laminate uniformity, reduced residual stresses, and ensured consistent fibre–matrix adhesion across all fabricated samples. It should also be noted that maintaining the same mould thickness does not necessarily imply identical internal fibre packing, fibre volume fraction, or porosity in all laminates. Since bark cloth and banana fibre differ in density and structural form, local consolidation characteristics may vary between configurations even under the same mould thickness and applied load. In the present study, thickness was controlled by mould geometry and compression pressure, whereas fibre volume fraction and void content were not independently measured.

#### Fiber weight fraction configuration

To systematically assess the hybrid effect of banana and bark cloth fibers, eight composite configurations were fabricated by varying the fiber weight fractions while maintaining a constant matrix-related content of 55 wt%, comprising 50 wt% epoxy + hardener and 5 wt% ATH. No separate weight-to-volume conversion procedure was used to define the final laminate compositions; instead, all formulations were established directly on a weight-percentage basis. Accordingly, only the banana and bark cloth fibre proportions were treated as experimental design variables, while the epoxy/ATH formulation was held constant to ensure that differences in performance could be attributed primarily to fibre hybridization rather than changes in matrix composition or additive loading. The composition of each configuration is presented in Table 3. In this study, ATH was intentionally fixed at 5 wt% as a baseline flame-retardant level; therefore, the GRA was used to optimize fibre-ratio configuration under a constant ATH loading rather than to optimize ATH content itself.

**Table 3:** Composite formulations of banana/bark cloth epoxy samples in weight percentages (wt%).

Composition No.	Banana Fiber (wt%)	Bark Cloth Fiber (wt%)	Epoxy + Hardener (wt%)	Fire Retardant (ATH) (wt%)
C1	10	35	50	5
C2	15	30	50	5
C3	20	25	50	5
C4	25	20	50	5
C5	30	15	50	5
C6	35	10	50	5
C7	40	5	50	5
C8	45	0	50	5

### Experimental plan

**Fig. 2:**
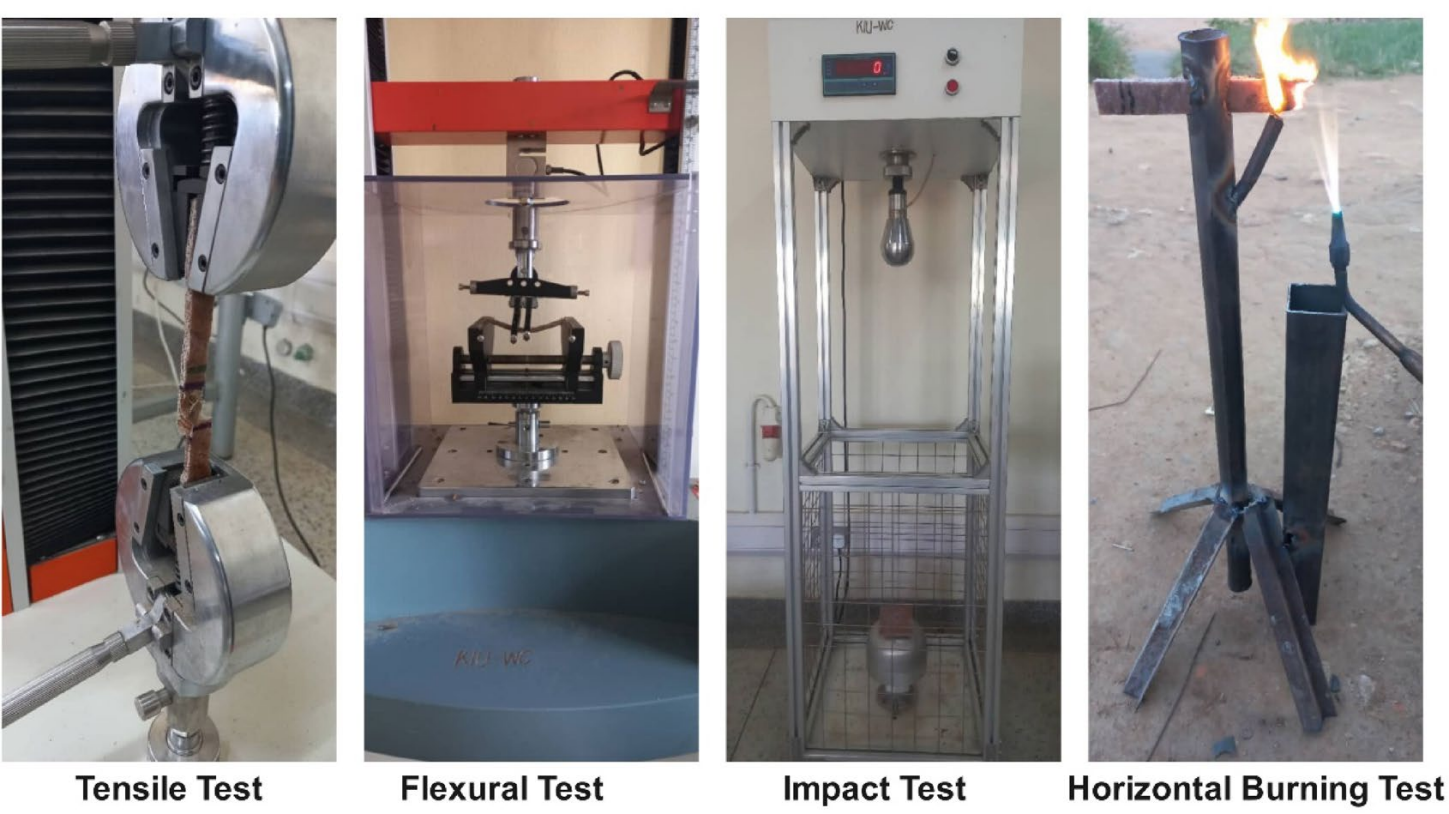
Photographs of experimental test setups used in the study.

To comprehensively evaluate the mechanical and flammability performance of the fabricated banana/bark cloth epoxy composites, standardized testing protocols were followed as per ASTM. These tests ensure repeatability, comparability, and benchmarking against international standards, which are crucial for evaluating the suitability of composites for automotive interior applications. For each composite composition, three replicate specimens were tested for every mechanical property and flammability performance. The reported results in this study represent the average values obtained from these three replicates, in line with standard practice to ensure accuracy and reliability. The photograph of various tests is depicted in Fig. 2.

#### Tensile testing

Tensile strength was assessed following the ASTM D638 standard, which specifies the method for determining tensile properties of reinforced and unreinforced plastics. This test measures the tensile strength, providing insights into the fiber-matrix adhesion and overall structural integrity of the composite^63^. The specimens were prepared in a Type I dog-bone geometry using a precision-cutting jig, with nominal dimensions of 165 mm in length, 13 mm in width, and 3 mm in thickness. The test was conducted using a Universal Testing Machine (Model: TM2101-T5, Haida International Equipment Co., Ltd., China) equipped with a 5 kN load cell and a Panasonic servo motor drive. The machine offers a crosshead speed range of 0.1–500 mm/min, load accuracy within ± 0.5% of full scale, and displacement accuracy of ± 0.1 mm, ensuring precise data acquisition. A crosshead speed of 5 mm/min was maintained for all tests to ensure quasi-static loading conditions. The integrated TM2101 software was used to capture real-time load–elongation data and automatically compute tensile strength and strain. This evaluation provided valuable insights into the fiber–matrix interfacial bonding, stiffness, and overall structural integrity of the banana/bark cloth hybrid epoxy composites.

#### Flexural testing

The flexural performance of the composites was evaluated in accordance with ASTM D790, which defines the procedure for determining the flexural strength and flexural modulus of unreinforced and reinforced plastics under three-point bending. Rectangular specimens of 127 mm × 12.7 mm × 3 mm were cut using a precision saw, and a span length of 48 mm (16 × thickness) was maintained between the supports. Testing was carried out on a Three-/Four-Point Bending Testing Machine (Model RT203B, China) equipped with interchangeable load-cell options of 500 N, 1 kN, 2 kN, and 5 kN. The machine offers a crosshead-speed capability of 0.05–500 mm min^− 1^. The tests were performed at a crosshead speed of 1.28 mm min^− 1^, as prescribed by ASTM D790, and the control software automatically recorded the force-deflection data.

This setup ensured precise control of bending stress and deflection, enabling reliable evaluation of flexural strength and modulus. The test provided key insights into the bending stiffness, interlaminar integrity, and structural reliability of the banana/bark-cloth hybrid epoxy composites properties particularly relevant to load-bearing panel structures in automotive interiors. The relevance of ASTM D790 in similar studies has been reaffirmed by Guo et al.^64^, who assessed polymer composites using this method and emphasized its importance in quantifying material flexural performance in structural applications.

#### Impact testing

The impact resistance of the fabricated composite samples was evaluated using the drop-weight impact method in accordance with ASTM D7136/D7136M-20, the standard procedure for assessing the damage resistance of fiber-reinforced composites to low-velocity impact events. Tests were carried out on a Drop Weight Impact Tester (Model AMQ, China) equipped with interchangeable impact and puncture hammers made of steel. The impact hammer weighed 5 ± 0.01 kg with a radius of 48 mm. The device incorporated a 20 kN force sensor (2–100% FS range, 1 N resolution) and operated at a maximum drop height of 1000 ± 5 mm, powered by a 220 V, 50 Hz supply. Test specimens measuring (150 mm × 100 mm × 3 mm) were clamped on a platform with a central circular cut-out to allow unobstructed deformation and penetration during impact. The absorbed energy was automatically recorded to determine the specific impact energy (J/g), enabling evaluation of energy absorption capacity, crack propagation behavior, and residual strength. The system provided accurate and repeatable measurements, ensuring reliable comparison among the hybrid composite configurations. This methodology closely simulates realistic low-velocity impacts commonly experienced by automotive interior panels, consistent with earlier studies on natural-fiber composites^58,65^.

#### Flame retardancy

Flame behavior was assessed using the ASTM D635 standard, which evaluates the rate of burning in a horizontal position of specimens measuring 125 mm × 13 mm × 3 mm, conditioned at 23 °C. The sample was ignited at one end using a standard Bunsen burner, and the burning rate (mm/s) was calculated by measuring the time taken for the flame to travel between marked intervals^66^. This method offers a practical and comparative metric for Horizontal Burning (HB) behavior and was used in this study as a preliminary laboratory-scale screening tool for comparing flame-spread performance among the composite formulations. However, ASTM D635 should not be interpreted as a direct substitute for automotive interior qualification standards such as FMVSS 302, which would be required for application-specific compliance assessment.

#### Water absorption testing

Water absorption tests were performed according to the procedure specified in ASTM D570, with minor modification to the specimen size due to fabrication constraints. Rectangular samples measuring 125 mm × 15 mm × 3 mm were used to maintain consistent geometry with other tests while preserving a similar surface-area-to-thickness ratio as recommended in the standard. All specimens were dried at 50 °C for 24 h, cooled in a desiccator, and weighed (W_1_) before immersion in distilled water at 23 ± 2 °C. The specimens were weighed at regular 24-hour intervals for 10 consecutive days, and the saturated weight (W_2_) recorded at the end of the tenth day was used to calculate the water absorption percentage (WA%) using Eq. (1).

1 $$\:\mathbf{W}\mathbf{a}\mathbf{t}\mathbf{e}\mathbf{r}\:\mathbf{A}\mathbf{b}\mathbf{s}\mathbf{o}\mathbf{r}\mathbf{p}\mathbf{t}\mathbf{i}\mathbf{o}\mathbf{n}\:\left(\mathbf{W}\mathbf{A}\right)\:\left(\mathrm{\%}\right)=\frac{\mathbf{W}2-\mathbf{W}1}{\mathbf{W}1}\times100$$

This method provides insights into fiber-matrix interface integrity and the resistance of the composite to moisture ingress, both of which directly affect mechanical durability^52^.

### Grey relational analysis method

In this research, Grey Relational Analysis (GRA) was employed as a robust multi-criteria decision-making tool to optimize the composite configurations based on multiple performance parameters, including tensile strength, flexural strength, impact resistance, flame retardancy, and water absorption. The method allowed for a systematic ranking of the hybrid banana/bark cloth epoxy composites by converting diverse property metrics into a unified performance index. Key steps such as data normalization (based on “larger-the-better” or “smaller-the-better” criteria), computation of grey relational coefficients (GRC), and derivation of the grey relational grade (GRG) were sequentially applied. These steps enabled the identification of the best-performing composite formulation under integrated mechanical and environmental performance goals. GRA provided efficient ranking on the development and characterization of barkcloth-velvetleaf fiber composites^67^. The mathematical formulations, normalization approach, and computational details used in the GRA are provided in the Supplementary Information.

### Characterization techniques

To comprehensively evaluate the microstructural, elemental, and chemical characteristics of the fabricated banana/bark cloth-reinforced epoxy composites, three key characterization techniques were employed: Scanning Electron Microscopy (SEM), Energy Dispersive X-ray Analysis (EDX), and Fourier Transform Infrared Spectroscopy (FTIR).

#### SEM with EDAX analysis

The surface morphology and fracture characteristics of the natural fibre composites (NFCs) were examined using a Scanning Electron Microscope (SEM, Zeiss EVO MA10, Carl Zeiss AG, Germany) operated at an accelerating voltage of 15 kV under high-vacuum conditions. Owing to the non-conductive nature of the epoxy–fiber composites, small specimens were sectioned from the fractured surfaces after tensile testing, mounted on aluminium stubs using carbon adhesive tape, and subsequently gold-sputter-coated for 60 s to enhance surface conductivity and prevent electrostatic charging during imaging. Micrographs were captured at magnifications ranging from 200× to 5000×, providing detailed insights into fiber pull-out, interfacial adhesion, void formation, and matrix continuity. SEM analysis revealed the underlying failure mechanisms under tensile and impact loading, allowing a direct microstructural correlation with the observed macroscopic mechanical behaviour. Elemental composition was further analyzed using Energy-Dispersive X-ray Spectroscopy (EDAX) integrated with the SEM to examine the chemical distribution at both the matrix and fiber interface zones. The EDAX spectra confirmed the presence of carbon (C) and oxygen (O) as the dominant elements from the organic matrix and fibres, along with aluminium (Al) from the ATH fire retardant and sodium (Na) originating from the NaOH treatment. This elemental evidence verified the successful surface modification and additive incorporation within the hybrid composite system, in agreement with previous reports^68^.

#### FTIR

Fourier Transform Infrared (FTIR) spectroscopy was conducted using a JASCO FT/IR-4600 Spectrometer (JASCO Corporation, Japan) to identify functional groups and investigate the chemical interactions between the banana/bark cloth fibers and the epoxy resin matrix. For analysis, powdered composite specimens (~ 50 μm particle size) were prepared by gently grinding small portions of the cured laminates to achieve a uniform surface suitable for measurement. Spectra were recorded in the 4000–400 cm^− 1^ range using the Attenuated Total Reflectance (ATR) mode at a spectral resolution of 4 cm^− 1^, averaged over 32 scans per sample. The FTIR analysis enabled detection of key absorption bands corresponding to –OH (hydroxyl), –COOH (carboxyl), –C = O (carbonyl), and –CH_2_ (methylene) groups, which are characteristic of lignocellulosic fibers and epoxy systems. Shifts in the intensity and position of these peaks provided clear evidence of surface modification following NaOH treatment, particularly the reduction of hydroxyl and carboxyl bands due to partial delignification and hemicellulose removal^68,69^. The emergence or enhancement of epoxy-related peaks further confirms interfacial interactions and hydrogen bonding between the treated fibers and matrix, as also reported in similar hybrid composite studies^68^. Collectively, the FTIR and SEM–EDAX analyses offered complementary insights into the microstructural integrity and interfacial chemistry of the hybrid composites, reinforcing the experimental findings on their mechanical and flammability performance.

## Results and discussion

The results obtained from the experimental investigation are presented and discussed in this section. The mechanical, flammability, and water absorption properties of the developed banana/bark cloth hybrid epoxy composites were analyzed to evaluate the influence of fibre composition and laminate architecture under fixed processing conditions. Each mechanical and flammability test was performed on three replicate specimens for every composite configuration, and the corresponding mean and standard deviation values are presented in Table 4. The Coefficient of Variation (CoV) was found to be within a maximum of 2%, confirming excellent repeatability and consistency of the experimental data. The detailed replicate-level results and statistical calculations are provided in the Supplementary Information for transparency and reproducibility. The calculated standard deviation values, with a maximum deviation of 6%, are consistent with those reported for hand lay-up processed natural fiber composites, indicating reliable data acquisition and minimal experimental variability. It is noted that certain properties, particularly flexural modulus and horizontal burning rate, exhibit relatively low standard deviation values compared to strength-based properties. This behavior is attributed to the high experimental repeatability achieved in the present study. All test specimens were cut from the same laminate plates, ensuring uniform fiber architecture, thickness, and resin distribution. In the case of flexural modulus, which is obtained from the initial linear slope of the load–deflection curve, the response was highly consistent across specimens, resulting in low variability. Similarly, the horizontal burning tests showed stable and repeatable flame-spread behavior under controlled laboratory conditions. These factors collectively contributed to the observed low scatter in these measurements. The subsequent subsections present a detailed analysis of the mechanical performance parameters, the outcomes of multi-response optimization, and the microstructural characterization of the optimal composite configuration.

**Table 4:** Mean and standard deviation of mechanical and flammability properties of banana/bark cloth epoxy composites.

Sample	Mean ± Standard Deviation				
	Tensile Strength (MPa)	Flexural Strength (MPa)	Flexural Modulus (MPa)	Specific Impact Energy (J/g)	Horizontal Burning (HB) Test (mm/s)
C1	4.693 ± 0.096	7.394 ± 0.168	9428.495 ± 6.3	111.842 ± 0.647	0.952 ± 0.012
C2	10.550 ± 0.181	15.787 ± 0.344	19747.108 ± 5.6	92.829 ± 0.585	0.952 ± 0.007
C3	17.876 ± 0.336	29.559 ± 0.495	36615.088 ± 6.4	80.636 ± 0.205	0.794 ± 0.009
C4	20.418 ± 0.210	32.157 ± 0.494	40354.485 ± 4.1	81.048 ± 0.198	1.111 ± 0.008
C5	20.904 ± 0.181	36.213 ± 0.206	45592.255 ± 2.2	98.511 ± 0.353	0.870 ± 0.004
C6	17.889 ± 0.302	33.803 ± 0.535	42839.635 ± 5.6	101.896 ± 0.222	1.000 ± 0.01
C7	28.284 ± 0.236	37.858 ± 0.603	48137.665 ± 5.4	106.921 ± 0.426	1.053 ± 0.006
C8	23.295 ± 0.168	29.346 ± 0.169	36469.383 ± 7.0	80.494 ± 0.240	0.833 ± 0.006

### Mechanical properties

#### Tensile strength

**Fig. 3:**
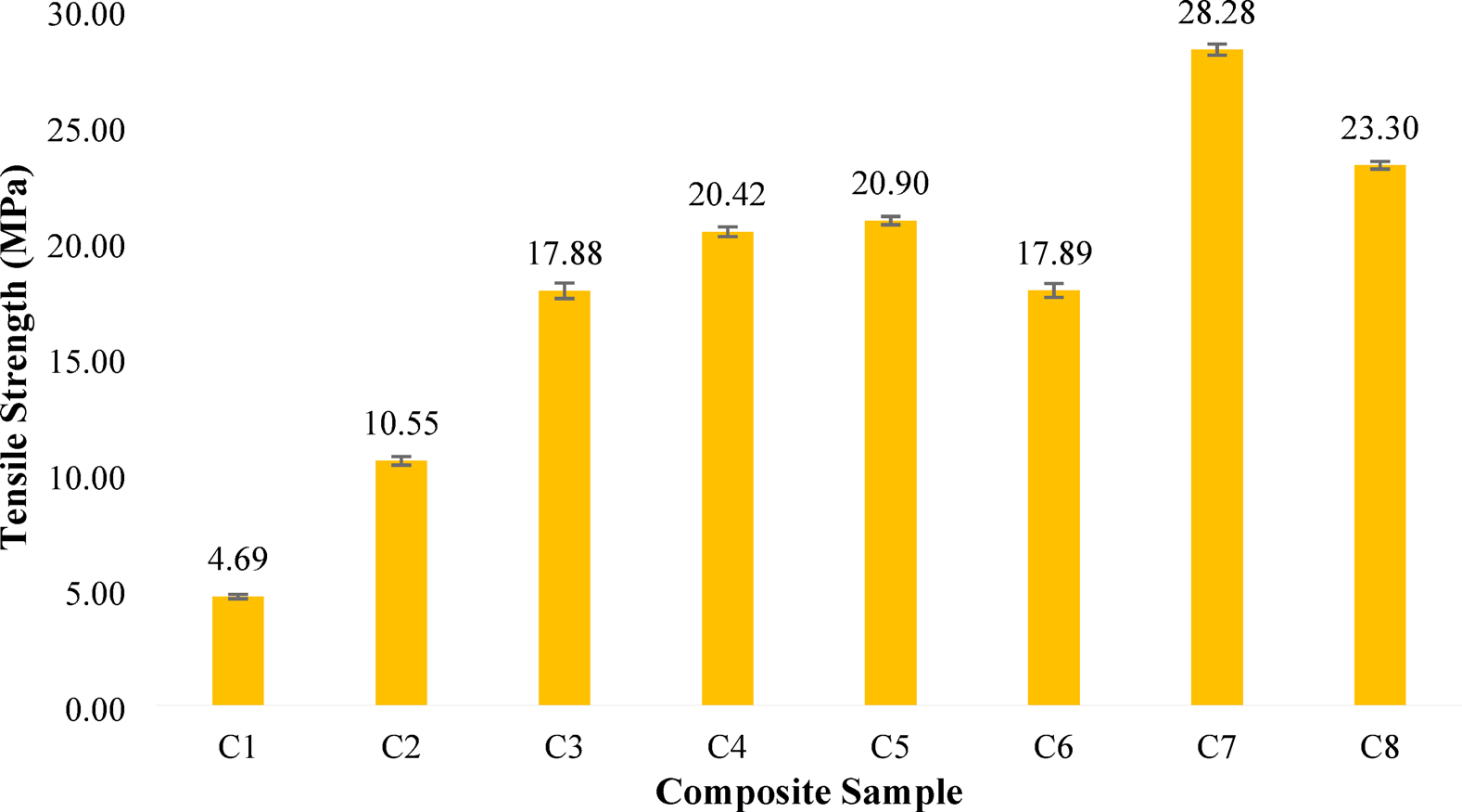
Tensile strength of banana/bark cloth composites. Error bars represent ± SD (*n* = 3).

The tensile strength results of the banana/bark cloth fiber-reinforced epoxy composites reveal a clear trend associated with changes in banana/bark cloth proportion together with the corresponding laminate stacking arrangement. As shown in Fig. 3, tensile strength increased steadily with the increment of banana fiber content, reaching a peak value of 28.28 MPa in Composition C7, which consisted of 40 wt% banana fiber and 5 wt% bark cloth fiber. This trend highlights the superior load-bearing capabilities of banana fiber when used as the primary reinforcement in an epoxy matrix. Banana fibers, being highly lignocellulosic with a strong microfibrillar angle and robust cellulose content^70^, contribute significantly to tensile load transfer when adequately dispersed and bonded with the matrix. The increase from 4.69 MPa in C1 to 20.90 MPa in C5 demonstrates the progressive reinforcement efficiency as banana content increases. Beyond this, the 5 wt% of bark cloth in C7 led to a substantial tensile performance boost.

On the other hand, bark cloth, although rich in lignin and relatively porous, did not contribute equally to the tensile strength. Its presence in higher proportions, as in C1–C3, resulted in lower tensile strength values, likely due to its lower stiffness and higher micro-void content, which may compromise the continuity of stress transfer under uniaxial loading. In contrast, compositions with balanced banana/bark cloth ratios, such as C3 and C4, exhibited moderate improvements but did not surpass the strength obtained with banana fiber dominance. Thus, the tensile response suggests that banana-rich laminates generally favour tensile enhancement in the present study; however, this trend should be interpreted together with the laminate architecture used in each configuration. The results are in agreement with recent literature on natural fiber-reinforced epoxy systems, where increasing banana fiber content improved tensile behavior due to better fiber alignment and interfacial bonding^58^.

#### Flexural strength and flexural modulus

**Fig. 4:**
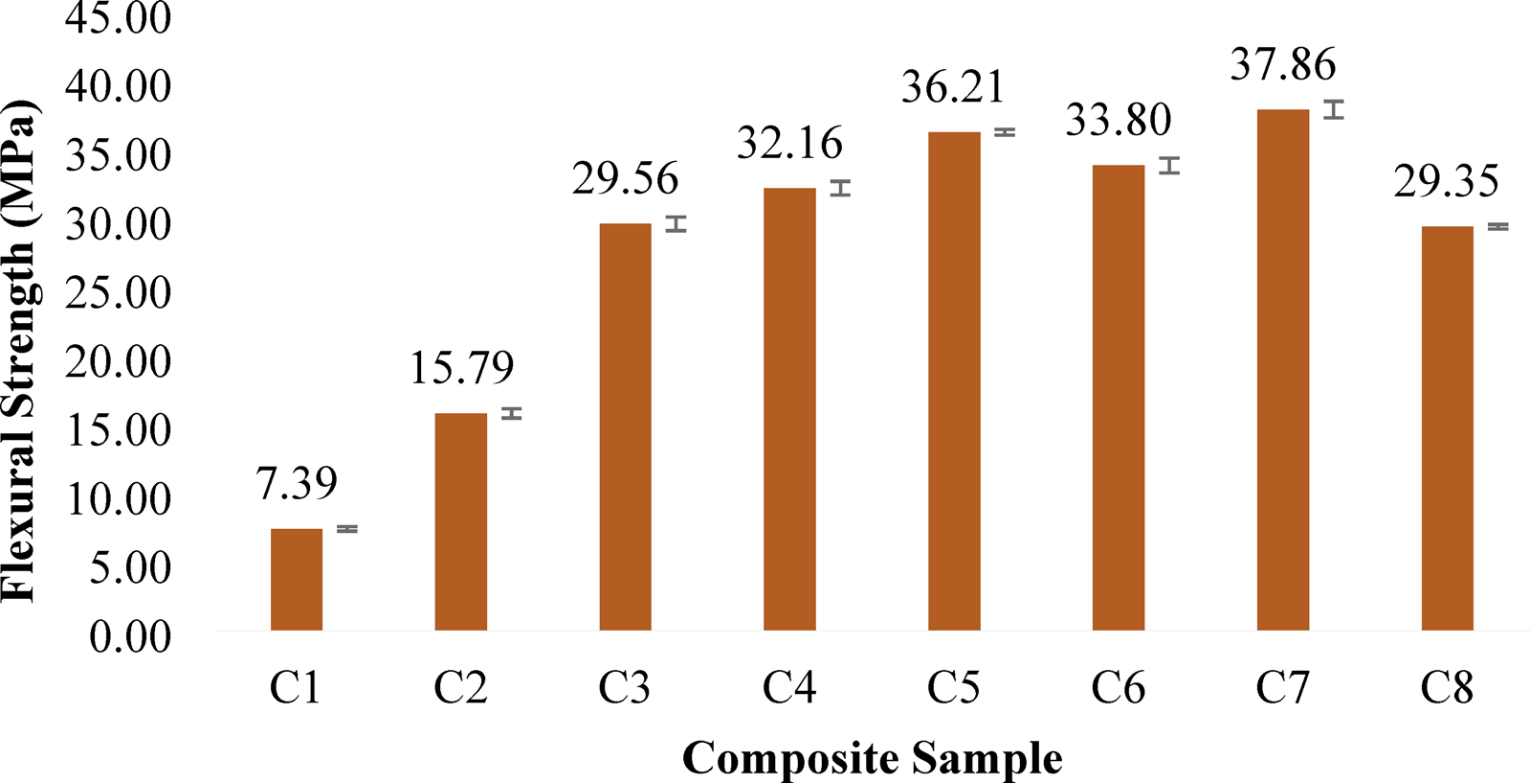
Flexural Strength of banana/bark cloth composites. Error bars represent ± SD (*n* = 3).

**Fig. 5:**
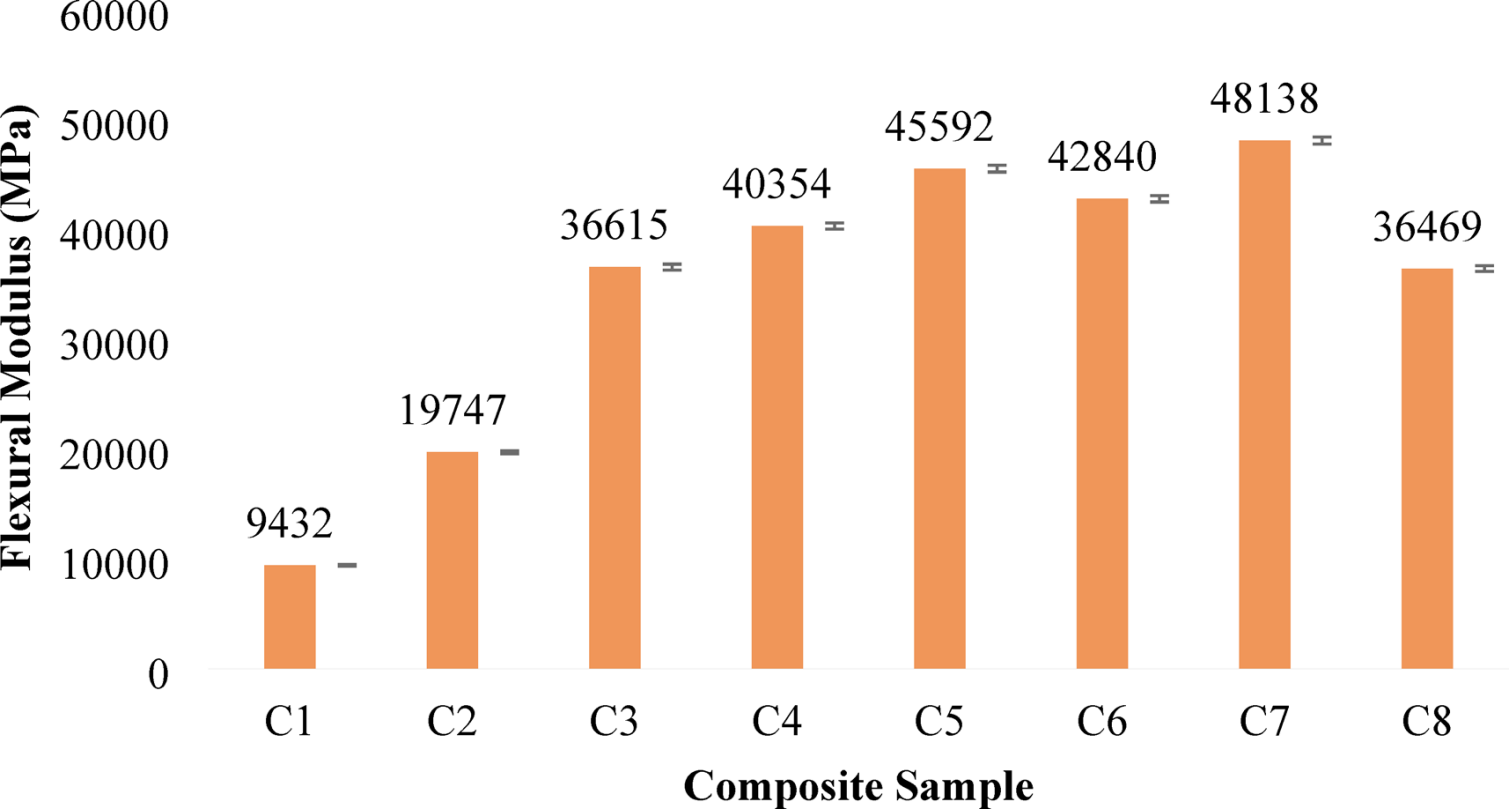
Flexural Modulus of banana/bark cloth composites. Error bars represent ± SD (*n* = 3).

The flexural performance of the banana/bark cloth fiber-reinforced epoxy composites reveals a nuanced relationship between fiber type, their relative proportions, and the resulting structural response under bending. As seen in Fig. 4, both the flexural strength and modulus in Fig. 5 exhibit an upward trend with increasing banana fiber content, reaching their respective maxima in Composition C7, which contains 40 wt% banana fiber and 5 wt% bark cloth fiber. C7 recorded a flexural strength of 37.86 MPa and a flexural modulus of 48,137.67 MPa, indicating excellent stiffness and bending resistance. This stiffness enhancement reflects the load-bearing potential and fiber packing efficiency of banana fibers. In contrast, C3 and C8, which feature either balanced or reduced banana content, displayed lower moduli, indicating reduced resistance to deformation under load.

The dominance of banana fiber in enhancing flexural strength can be attributed to its higher tensile modulus, improved interfacial bonding due to alkaline treatment, and its aligned microstructure, which efficiently transfers bending stresses across the fiber–matrix interface. As the banana content increased progressively from C1 to C7, the composite demonstrated improved ability to withstand bending forces. This improvement is consistent with findings by Senthamizh Selvan & I. S^71^.,, who reported that carbon or natural fibers with strong matrix adhesion and high aspect ratios contribute significantly to flexural load resistance. Meanwhile, bark cloth, though beneficial for fire retardancy and impact dissipation due to its crimped and irregular surface morphology, appears less effective under flexural stress. For example, C1 showed the lowest flexural strength and flexural modulus of 7.39 MPa, likely due to the limited mechanical contribution of bark cloth fibers in resisting bending-induced tensile and compressive zones. However, intermediate compositions like C4 and C5 yielded appreciable flexural strength values of 32.16 MPa and 36.21 MPa, respectively, suggesting a beneficial synergy when banana fibers are dominant and bark cloth content remains moderate.

The mechanical behavior revealed here aligns with composite design principles that fibers with higher Young’s modulus and aligned orientation under load dominate the flexural response. Moreover, the pre-treatment with NaOH likely facilitated improved stress transfer across the fiber–matrix interface by removing hemicellulose and increasing surface roughness effects supported in literature dealing with natural fiber composites optimized for automotive and structural use^58^.

Thus, the results effectively illustrate this compositional dependency, where flexural performance is maximized with a high banana-to-bark cloth fiber ratio. The results confirm that banana fibers act as the structural backbone of the hybrid composite, while bark cloth must be judiciously balanced to avoid diluting the mechanical integrity.

#### Specific impact energy

**Fig. 6:**
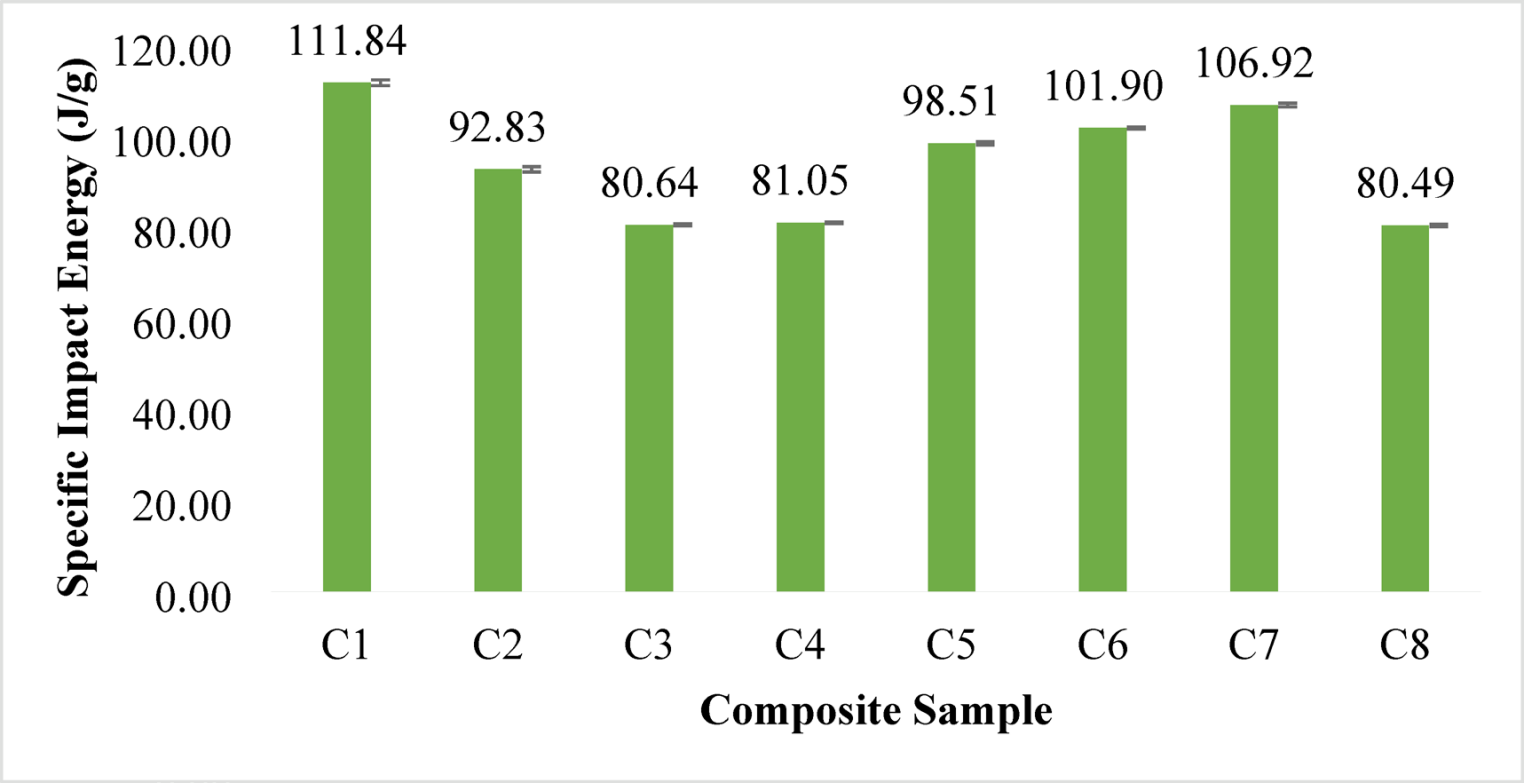
Specific Impact Energy of banana/bark cloth composites. Error bars represent ± SD (*n* = 3).

The specific impact energy results provide critical insights into the energy absorption capability of the banana/bark cloth hybrid composites under dynamic loading conditions. This property is especially relevant in automotive interior applications where materials must withstand accidental knocks, low-velocity collisions, or mechanical shocks without catastrophic failure. As presented in Fig. 6, specific impact energy varies across compositions, with noticeable dependence on fiber ratios, particularly the balance between banana and bark cloth fibers. The highest value of 111.84 J/g was recorded for Composition C1, while C6 and C7 also showed elevated values of 101.90 J/g and 106.92 J/g, respectively. These results suggest that both high bark cloth and high banana contents can independently contribute to improved impact resistance, albeit through different mechanisms. Bark cloth’s role in enhancing impact energy is primarily due to its loose, porous structure and laminated configuration, which facilitates energy dissipation through interlayer friction, fiber pull-out, and localized deformation zones. This effect is evident in C1 and C2, where high bark cloth content contributes to better energy absorption despite their relatively low mechanical stiffness. This observation is consistent with the findings of Jen et al.^72^, who demonstrated that hybridization of natural fibers in different orientations enhanced impact resistance due to improved crack deflection and damping response.

Conversely, the high specific impact energy in C6 and C7, where banana fiber dominates, can be attributed to banana’s inherent toughness and interfacial bonding strength. In these samples, the high tensile and flexural properties work synergistically to resist the propagation of impact-induced damage. This dual behavior suggests that both banana and bark cloth fibers contribute positively to impact performance, albeit through complementary mechanisms: banana fibers offer structural toughness, and bark cloth offers enhanced energy dissipation.

Interestingly, intermediate compositions, such as C3 and C8, exhibit lower specific impact energies (80.64 J/g and 80.49 J/g), indicating that certain hybrid ratios may not yield optimal fiber-matrix interactions or may suffer from microstructural inefficiencies. These configurations could potentially introduce voids, weak interfaces, or heterogeneous stress distribution zones, which compromise energy absorption. The specific impact energy results underline the non-linear relationship between fiber ratio and impact resistance. They reveal that both extremes of banana or bark cloth fiber content can be beneficial when supported by optimized matrix bonding and fiber distribution. The drop weight test method, chosen for its real-world applicability.

#### Horizontal burning

**Fig. 7:**
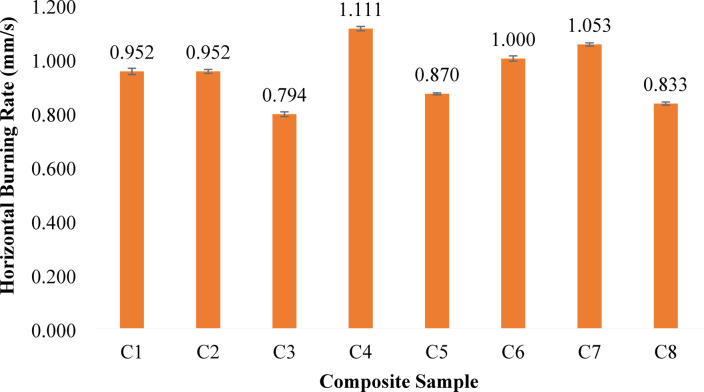
Horizontal Burning of banana/bark cloth composites. Error bars represent ± SD (*n* = 3).

The horizontal burning (HB) test shown in Fig. 7 provides important insight into how the composites respond to fire, which is especially relevant for applications like automotive interiors where fire safety standards are strict. The results clearly show that the burning rate depends on the type and ratio of fibers used. Among all samples, C3 recorded the lowest burning rate at 0.794 mm/s, showing the best flame resistance. C8 (0.833 mm/s) and C5 (0.870 mm/s) also performed well, indicating that low burning rate cannot be attributed to bark-cloth proportion alone. This effect is likely due to the bark cloth’s natural ability to form a protective char layer and provide thermal insulation. Similar observations have been reported by Zhang et al.^73^, who found that natural fibers rich in carbon and lignin tend to enhance flame resistance through char formation during burning.

On the other hand, C4 showed the highest burning rate at 1.111 mm/s, followed by C7 (1.053 mm/s) and C6 (1.000 mm/s). These samples, which contain a higher amount of banana fiber, burned more quickly probably because banana fibers have more cellulose and lower density, making them easier to ignite and sustain combustion. This suggests a trade-off in some configurations; however, flame resistance does not follow a simple monotonic trend with banana or bark-cloth content alone.

All composites included 5 wt% of ATH as a flame retardant. Since this amount was kept constant across all samples, the variation in burning rates mainly reflects differences in laminate design, including fibre composition and stacking arrangement. As noted by Nakhate et al.^74^,, ATH enhances fire performance by releasing water vapor and forming a protective layer when heated, though its effectiveness depends on how it interacts with the fiber and matrix.

Notably, hybrid samples like C3 and C8, where banana and bark cloth fibers are used in balanced ratios, achieve a good compromise lower flammability without losing too much strength. This demonstrates a synergistic effect between the two fibers: favourable flame behaviour appears to result from the combined effects of fibre type, hybrid ratio, laminate architecture, and matrix interaction. Overall, the HB test results in Fig. 7 highlight how thoughtful fiber selection and hybridization can create composites that are both strong and fire-safe. Incorporating bark cloth fibers strategically appears to be a key step toward achieving safer, more sustainable materials.

#### Water absorption test

**Fig. 8:**
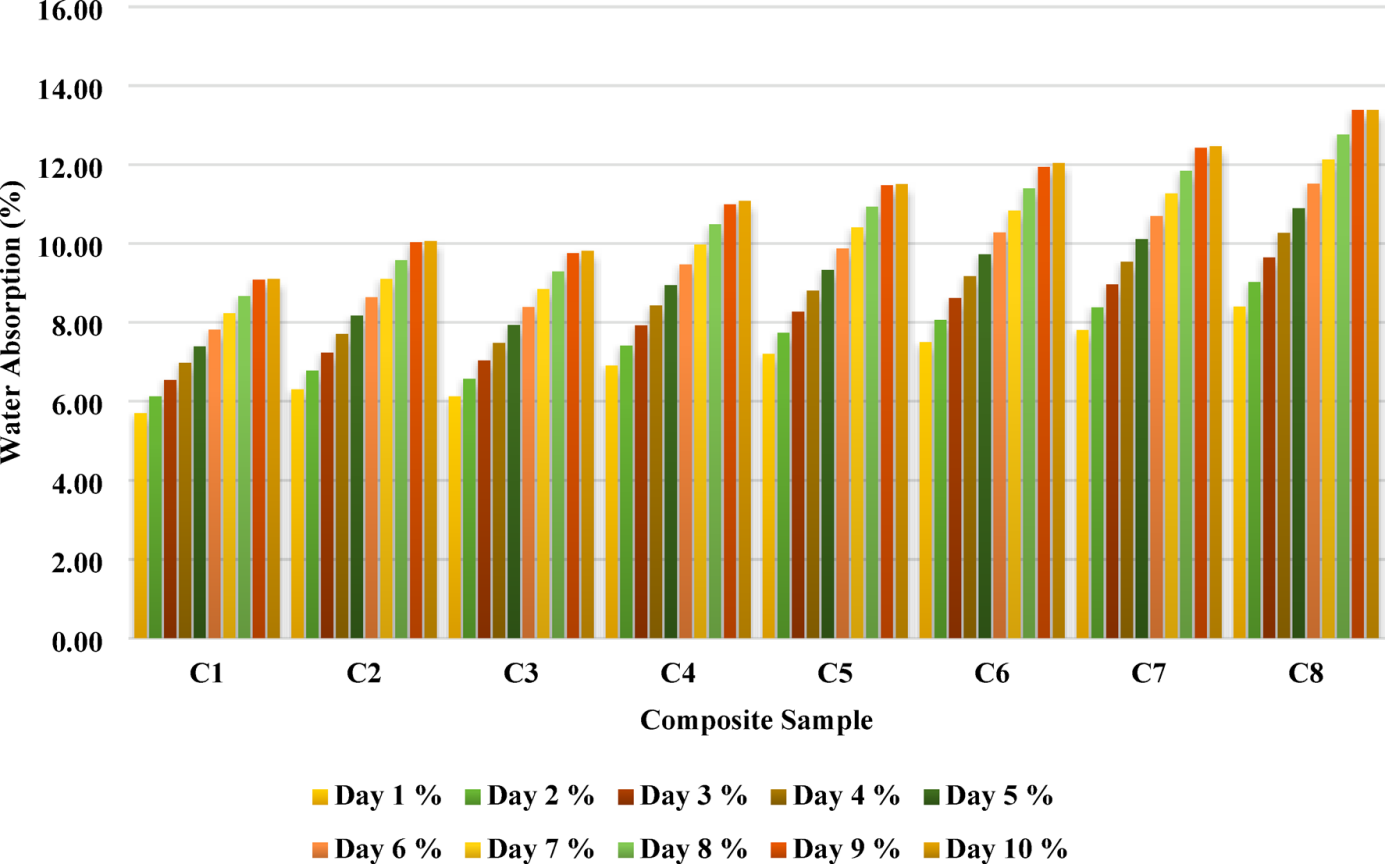
Water Absorption test.

The water absorption behavior of the banana/bark cloth fiber-reinforced epoxy composites over a 10-day immersion period reveals critical insights into the hydrophilic tendencies of each composite formulation. As shown in Fig. 8, the percentage of water absorbed increases steadily across all composite samples from Day 1 to Day 10, with higher banana fiber content clearly resulting in higher overall water uptake. This trend aligns with well-established findings that natural fibers, particularly banana, are inherently hydrophilic due to the presence of hydroxyl groups in their cellulose and hemicellulose structures, which readily interact with water molecules via hydrogen bonding.

Among the tested samples, C1 exhibited the lowest water absorption, reaching only 9.10% by Day 10. This is likely due to the predominance of bark cloth fiber, which possesses a more compact laminar structure, lower surface roughness, and potentially more lignin content, thereby resisting water ingress more effectively. In contrast, C8 absorbed the most water, reaching 13.39% by Day 10. The absence of bark cloth in this sample, combined with the high concentration of banana fiber, increased porosity, and the number of hydrophilic sites, thereby promoting water uptake.

The rate of absorption is almost linear for all samples during the initial days (Days 1–3), likely due to rapid diffusion into the accessible pores and capillaries of the fiber-matrix network. From Day 4 onward, the absorption rate becomes more composition-dependent. In banana-rich composites such as C6, C7, and C8, the absorption curve steepens, indicating increased fiber saturation and possibly the initiation of matrix swelling or microcracking. Conversely, composites with balanced or bark-rich formulations (C2–C4) show a more gradual rise, indicating that the matrix-fiber interface remained relatively intact, limiting capillary water ingress.

These findings underscore the importance of hybridizing banana with bark cloth fiber to balance mechanical properties and moisture resistance. While banana fibers enhance tensile and flexural performance due to their stiffness and surface area, they also elevate the composite’s vulnerability to moisture-related degradation. Bark cloth fibers act as a mitigating agent, slowing water diffusion and contributing to dimensional stability. Composites such as C3 and C4 emerge as optimal formulations, where water absorption at Day 10 remains below 11.1%, a threshold considered acceptable for many automotive interior applications.

The water-absorption behavior of natural fiber composites is critically influenced by the hydrophilic nature of plant fibers, especially banana fiber and bark cloth, which are rich in hemicellulose and pectin. The presented data demonstrates a clear increase in water uptake over time for all samples (C1–C8), with a stronger trend associated with increasing banana fiber content, culminating in maximum absorption in C8 (13.39% on Day 10).

Motaleb et al.^39^ evaluated water absorption in banana fiber composites using various treatments. They found that untreated epoxy composites absorbed significantly more water, whereas alkali and water-repellent treatments reduced water uptake by up to 63%. In their work, OB (outer bark)-based composites exhibited higher absorption due to structural looseness and porosity. Our results are consistent with their findings, particularly in C7 and C8, where the dominant banana content correlates with higher uptake. Jeevan Rao et al.^38^ investigated alkali-treated banana and *Careya* fiber composites, coated with PLA. They demonstrated that such treatments could reduce water absorption by nearly 48%, further emphasizing the importance of NaOH treatment and polymer coatings in minimizing hydrophilicity. This aligns with the need for fiber treatment in future studies on similar banana/bark cloth systems.

The experimental data align well with the published literature, indicating that banana fiber’s high cellulose and hemicellulose content leads to greater moisture absorption due to its porous microstructure. In contrast, bark cloth fibers, being more lignified, offer slightly better water resistance. The composite formulations from C1 to C8, where banana fiber progressively replaces bark cloth, highlight a clear trade-off: improved mechanical strength but reduced moisture resistance.

### Grey relational analysis

Building upon the diverse mechanical, impact, and flame-retardant behaviors observed across the different banana/bark cloth fiber composite configurations, it becomes evident that no single composition inherently excels across all performance metrics. While banana-rich composites demonstrated superior tensile and flexural strength, the inclusion of bark cloth significantly enhanced flame resistance, and intermediate hybrids showed optimal impact behavior. This multifaceted performance profile highlights the inherent trade-offs involved in natural fiber hybridization and underscores the need for a comprehensive evaluation framework. To address this, Grey Relational Analysis (GRA) was employed in this study as a powerful decision-making tool, capable of integrating multiple performance criteria, such as tensile strength, flexural properties, impact energy, and horizontal burning rate, into a single relational grade. By applying GRA, each composite configuration can be ranked holistically, enabling the identification of an optimal fiber composition that balances structural performance, safety, and durability. This approach not only bridges the gap between experimental complexity and practical material selection but also provides a reproducible, systematic pathway for optimizing sustainable composites for automotive interior applications.

**Table 5:** Grey Relational results.

Sample	Normalized Data				Deviation Sequence				Grey Relational Coefficient				Grey Relational Grade (GRG)	Rank
	TS	FS	SIE	HB	TS	FS	SIE	HB	TS	FS	SIE	HB		
C1	0.00	0.00	1.00	0.50	1.00	1.00	0.00	0.50	0.33	0.33	1.00	0.50	0.54	6
C2	0.25	0.28	0.39	0.50	0.75	0.72	0.61	0.50	0.40	0.41	0.45	0.50	0.44	8
C3	0.56	0.73	0.00	1.00	0.44	0.27	1.00	0.00	0.53	0.65	0.33	1.00	0.63	3
C4	0.67	0.81	0.02	0.00	0.33	0.19	0.98	1.00	0.60	0.73	0.34	0.33	0.50	7
C5	0.69	0.95	0.57	0.76	0.31	0.05	0.43	0.24	0.62	0.90	0.54	0.68	0.68	2
C6	0.56	0.87	0.68	0.35	0.44	0.13	0.32	0.65	0.53	0.79	0.61	0.43	0.59	5
C7	1.00	1.00	0.84	0.18	0.00	0.00	0.16	0.82	1.00	1.00	0.76	0.38	0.79	1
C8	0.79	0.72	0.00	0.88	0.21	0.28	1.00	0.13	0.70	0.64	0.33	0.80	0.62	4

The HB values reported in the normalized-data Table 5 are dimensionless normalized scores used for GRA ranking and should not be interpreted as the actual measured horizontal burning rates (mm/s). It should be noted that ATH was maintained constant for all samples; therefore, the GRA ranking is intended to compare the relative influence of banana/bark cloth hybridization on the combined responses under a fixed flame-retardant baseline. The Grey Relational Analysis (GRA) summarized in Table 5 provides a consolidated performance evaluation of eight hybrid banana/bark cloth fiber-reinforced epoxy composites by integrating four key criteria Tensile Strength (TS), Flexural Strength (FS), Specific Impact Energy (SIE), and Horizontal Burning (HB) rate into a single performance index using Grey Relational Grade (GRG). In this multi-response optimization framework, TS, FS, and SIE were classified under the “larger-the-better” category, given their importance in ensuring structural integrity and energy dissipation, while HB, which reflects flammability, was treated as “smaller-the-better” to prioritize fire safety. Equal weightage was assigned to each property to avoid bias, enabling a balanced performance assessment. The normalization data reveal that C7 stands out with normalized values of 1.00 in TS and FS, and 0.84 in SIE, indicating exceptional structural robustness while maintaining a moderately low HB value of 0.18, confirming its flame-retardant potential. This aligns well with prior mechanical and impact testing, in which C7 (40 wt% banana, 5 wt% bark cloth) consistently demonstrated high tensile and flexural strengths (28.28 MPa and 37.86 MPa, respectively), strong energy absorption (106.921 J/g), and acceptable flame behavior (1.053 mm/s). C5 ranked second with a GRG of 0.68, demonstrating robust flexural performance (36.213 MPa) and good tensile strength (20.904 MPa), thanks to its balanced fiber hybridization. On the other hand, C3 ranked third (GRG = 0.63), despite having the lowest normalized SIE (0.00) and the highest normalized HB score (1.00), due to its decent tensile and flexural scores (17.876 MPa and 29.559 MPa). Interestingly, C1, with minimal banana content (10 wt%), had the highest normalized SIE (1.00) but very poor TS and FS (0.00 each), suggesting that bark cloth enhances impact resistance while compromising structural rigidity, ranking it 6th. The poorest-performing sample, C2 (GRG = 0.44), combined weak TS and FS with moderate SIE and HB values, highlighting that low mechanical and thermal synergy leads to suboptimal composites. C4, although the lowest normalized HB score (0.00), scored lower in mechanical metrics, which pushed its rank to 7. This performance landscape reveals a distinct trade-off pattern between stiffness and flame resistance. Increasing banana fiber content improves tensile and flexural properties by enhancing fiber pull-out resistance and alignment, while bark cloth enhances energy absorption and fire-retardancy through its loose weave and char-forming ability. However, excessive bark cloth (as in C1) or banana-only compositions (as in C8) tend to skew the balance, affirming the necessity of controlled hybridization. The minor non-linear trend observed in the GRA results, where C3 slightly outperformed C4 despite similar compositions, is attributed to the integrated multi-response weighting used in Grey Relational Analysis. C3 exhibited improved flammability behaviour (lower HB rate) and a balanced SIE, resulting in a higher overall grey relational grade than C4. Since GRA simultaneously considers multiple normalized responses, small advantages in one parameter can shift the overall ranking. These deviations are within the expected experimental variation and do not influence the general optimization pattern. The GRA rankings strongly align with prior interpretations of the individual test results, establishing C7 as the optimal formulation that offers the best compromise across strength, impact resistance, and fire performance for automotive interior applications.

### Characterization of optimal sample

#### SEM analysis

**Fig. 9:**
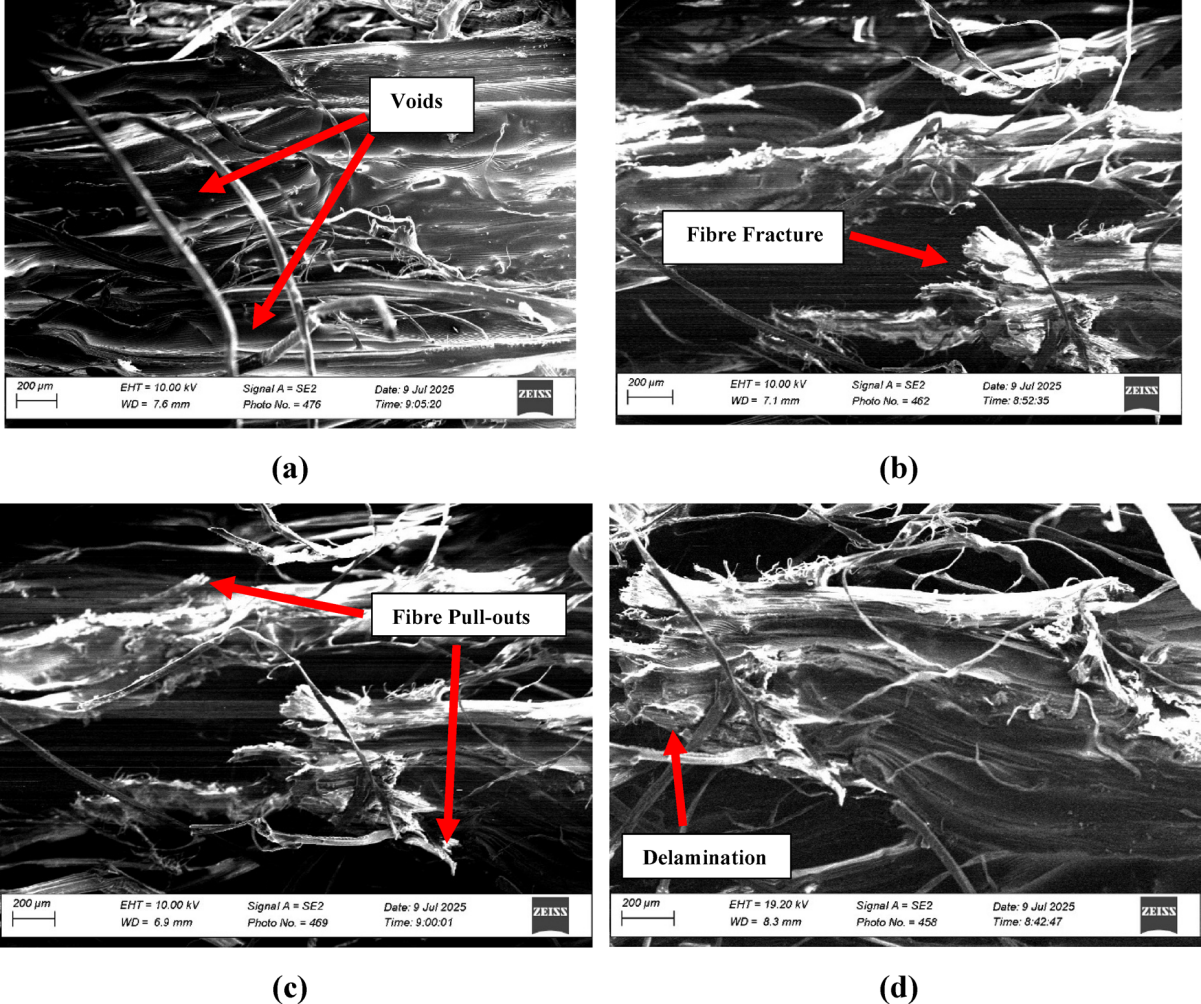
SEM of the fractured surface of the optimized hybrid composite (Sample C7: 40 wt% banana fiber + 5 wt% bark cloth fiber + 50 wt% epoxy + 5 wt% ATH) after tensile failure. Distinct morphological features include strong fiber–matrix interfacial bonding, limited fiber pull-out, and cohesive fracture zones, consistent with improved load transfer and good resin wetting in the imaged regions. All samples were gold-sputter-coated before imaging to eliminate charging artifacts.

The Scanning Electron Microscopy (SEM) micrographs shown in Fig. 9a–d depict the surface and fractured morphologies of the optimized hybrid composite sample (C7), comprising 40 wt% banana fiber, 5 wt% bark cloth, 50 wt% epoxy-hardener system, and 5 wt% ATH fire retardants. These micrographs were acquired to reveal microstructural features, including fiber dispersion, matrix-fiber interaction, interfacial bonding, and failure mechanisms, which collectively explain the superior mechanical and functional performance of C7 observed in previous tests. The SEM micrographs of the examined regions showed relatively good fibre-matrix continuity, limited fibre pull-out, and only small localized void features. These observations are qualitative and should not be interpreted as a bulk quantitative measure of laminate porosity.

In Fig. 9a, the micrograph depicts a relatively uniform dispersion of banana fibers within the epoxy matrix, with minimal fiber pull-out and voids. The well-embedded fibers indicate good wettability of the epoxy over the treated banana fiber surfaces, suggesting that alkali treatment with NaOH effectively removed surface impurities, enhanced surface roughness, and increased the number of hydroxyl groups that promote interfacial adhesion. These features may contribute to the higher tensile and flexural strengths recorded for C7 (28.28 MPa and 37.86 MPa, respectively) by reducing fibre pull-out and enabling more effective load transfer. Moreover, the interlocked fiber-matrix interface supports load transfer under tensile and bending loads, consistent with other studies that associate better interface bonding with enhanced modulus and stiffness.

Figure 9b provides evidence of bark cloth integration, were finer, networked fibrils can be seen bridging the epoxy matrix. The loosely woven, fibrous morphology of bark cloth contributes to crack arrest and energy dissipation during impact, helping explain the high specific impact energy of 106.92 J/g observed in C7. The image also shows thin lamellae of bark fibers adhered to the matrix, suggesting partial mechanical anchoring that helps absorb energy by delocalizing stress and promoting progressive failure rather than brittle fracture.

In Fig. 9c, the fracture surface of the composite under tensile load reveals fiber breakage zones interspersed with matrix cracking. The broken fiber ends appear jagged and rough, indicating fiber rupture rather than pull-out, a sign of strong interfacial bonding. Some matrix-rich regions are visible between the banana fibers, indicating good resin flow during fabrication and further contributing to uniform stress distribution. The crack path appears tortuous, a hallmark of energy-intensive failure and strong mechanical interlocking, reinforcing the composite’s high tensile strength. The absence of large delamination’s or macro-voids supports the conclusion that the composite was well cured and consolidated during compression moulding.

Figure 9d highlights interfacial fibrillation and matrix deformation, with several banana and bark fiber bundles intertwined with the matrix. This fiber interpenetration acts as a reinforcing network, restricting matrix mobility and enhancing stiffness. The matrix is observed to tightly conform to the fiber perimeters, forming a well-defined interface, particularly critical for stress transfer in bending and impact conditions. Additionally, ATH particles (though not clearly resolved due to scale) are expected to be distributed in these zones, contributing to flame retardancy by promoting char formation and reducing thermal decomposition during heat exposure.

A clear correlation between the SEM-observed surface morphology and the GRA ranking was evident. The alkali-treated banana fibers in the top-ranked composite (C7) displayed enhanced surface roughness and strong interfacial bonding, which facilitated efficient stress transfer and superior overall performance. In contrast, bark cloth–rich composites with smoother, laminar surfaces (e.g., C1–C3) exhibited weaker fiber–matrix adhesion, consistent with their comparatively lower GRA grades. The SEM micrographs further reveal that the optimal composite (C7) possesses a well-developed fiber–matrix architecture characterized by enhanced adhesion, reduced porosity, and integrated microstructural integrity. These morphological attributes substantiate the relationship between fiber surface topography and the multi-response optimization outcomes. Collectively, the SEM evidence rationalizes the multi-functional excellence of C7 as identified through Grey Relational Analysis and experimental testing, confirming that the synergy among banana fiber reinforcement for mechanical strength, bark cloth for impact resistance, and ATH for flame retardancy is structurally validated at the micro-level.

#### EDAX spectrum

**Fig. 10:**
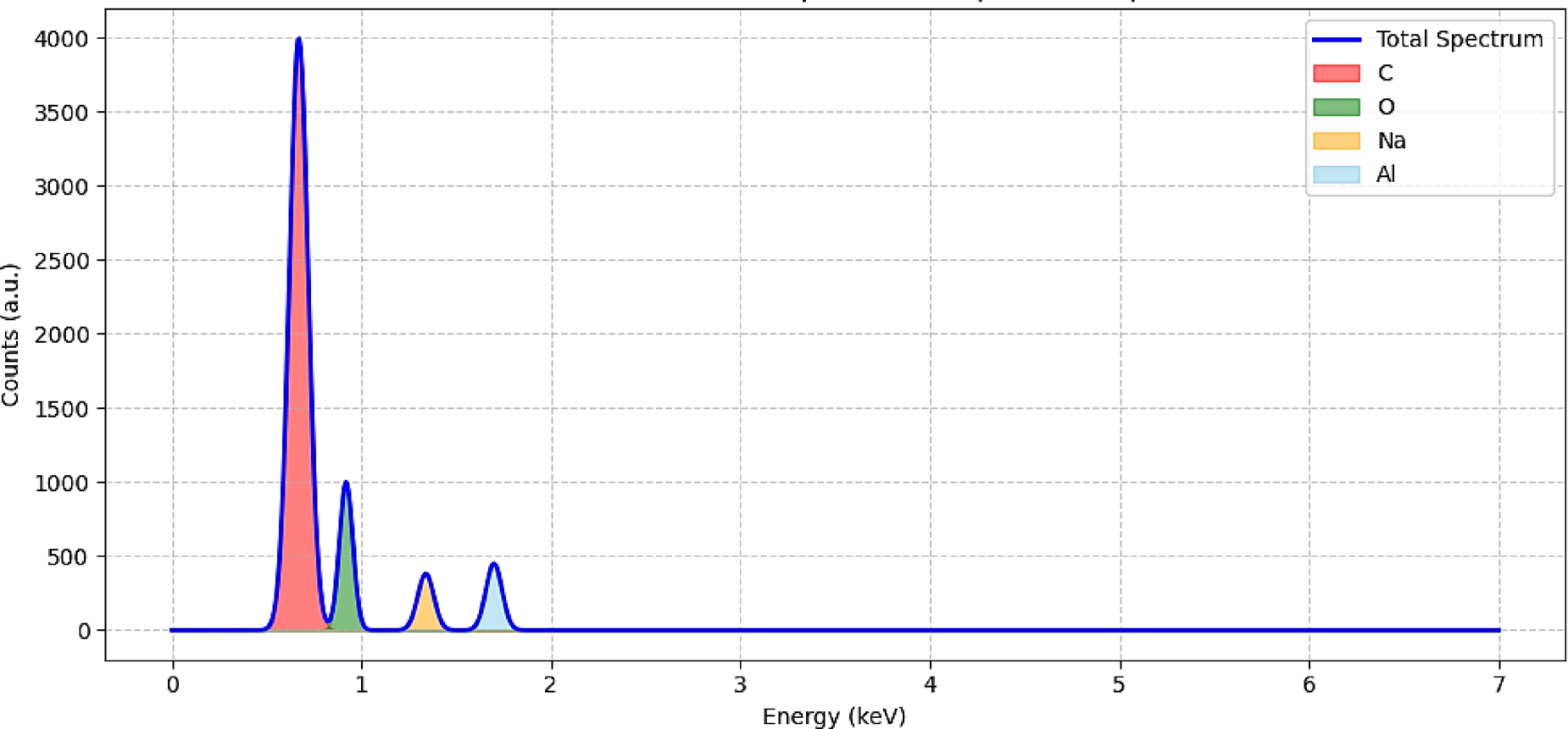
Energy-Dispersive X-ray Analysis (EDAX) spectrum of the optimized hybrid composite (Sample C7: 40 wt% banana fiber + 5 wt% bark cloth fiber + 50 wt% epoxy + 5 wt% ATH).

**Table 6:** EDAX Spectrum Quantification.

Element	Weight%	Atomic %	Net Intensity
C	73.68	79.09	458.34
O	25.26	20.35	97.68
Na	0.56	0.31	3.77
Al	0.5	0.24	4.37

The Energy-Dispersive X-ray Spectroscopy (EDX) analysis provides detailed insight into the elemental composition of the optimal composite sample (C7), and the results are presented in both the spectrum shown in Fig. 10 and the quantification data in Table 6. The EDAX spectrum confirms the presence of four main elements: carbon (C), oxygen (O), sodium (Na), and aluminium (Al), each with characteristic K-lines. Carbon and oxygen dominate the spectrum, comprising 73.68 wt% and 25.26 wt%, respectively, which is consistent with the organic, lignocellulosic structure of the banana and bark cloth fibers and the oxygen-rich epoxy matrix. These elements represent the bulk material composition and affirm successful fabrication. Sodium and aluminium, though minor (0.56 wt% and 0.50 wt%, respectively), are particularly significant as they validate the inclusion of processing agents NaOH for fiber treatment and ATH for flame retardancy. The atomic percentages and corresponding low error rates for C and O indicate robust signal acquisition and quantification fidelity, while slightly higher error margins for Na and Al reflect their trace presence but do not undermine their significance. The net intensity values further support this trend, with C yielding the highest counts (458.34), followed by O (97.68), in alignment with expected composition. Overall, the data confirm that the composite is chemically integrated as intended, with both functional treatment agents and matrix-fiber compatibility clearly evident in elemental mapping. This table, in conjunction with the spectral graph, supports the conclusions drawn from morphological and thermal analysis.

#### FTIR spectra

**Fig. 11:**
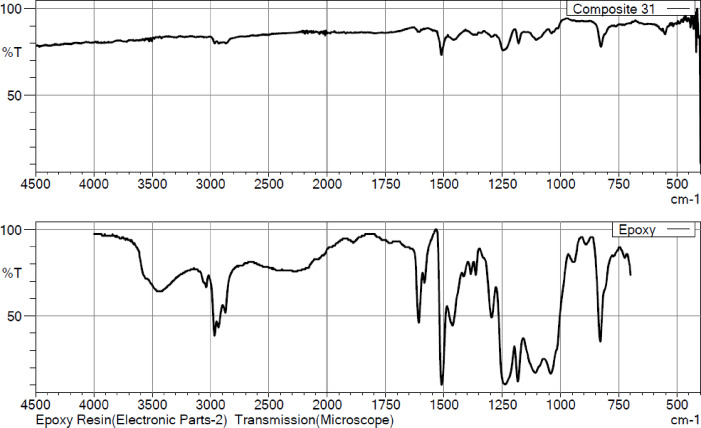
Fourier Transform Infrared (FTIR) spectra of (**a**) neat epoxy resin and (**b**) optimized hybrid composite (Sample C7: 40 wt% banana fiber + 5 wt% bark cloth fiber + 50 wt% epoxy + 5 wt% ATH) recorded using a JASCO FT/IR-4600 spectrometer in ATR mode (resolution 4 cm^− 1^, 32 scans).

The FTIR spectra presented in Fig. 11 compare the functional group composition of the optimal banana/bark cloth fiber-reinforced epoxy composite (top spectrum) with that of the neat epoxy resin (bottom spectrum). The spectrum of the neat epoxy exhibits characteristic peaks at ~ 3400 cm^− 1^ (O–H stretching), ~ 2920 cm^− 1^ and ~ 2850 cm^− 1^ (C–H aliphatic stretching), ~ 1600–1500 cm^− 1^ (aromatic ring vibrations), and sharp bands near ~ 1240 cm^− 1^ and ~ 830 cm^− 1^, which are attributed to epoxy ring breathing and C–O–C stretching vibrations. In the composite sample, these epoxy-related peaks appear slightly diminished and shifted, indicating partial chemical interaction between the epoxy matrix and natural fiber constituents. Notably, a broadening around ~ 3300 cm^− 1^ is seen in the composite, which may correspond to enhanced intermolecular hydrogen bonding, especially due to residual hydroxyl groups from banana and bark cloth fibers. The relatively weaker absorption in the fingerprint region (1200–700 cm^− 1^) in the composite suggests some degree of network rearrangement or crosslinking modifications. Moreover, the reduced transmittance near ~ 1740 cm^− 1^, typically associated with carbonyl (C = O) stretching, confirms the integration of cellulose-based fibers into the matrix. Overall, the FTIR spectrum of the composite reveals the retention of key epoxy characteristics, along with new or shifted peaks indicative of successful interfacial bonding and molecular compatibility between the hydrophilic natural fibers and the hydrophobic epoxy matrix. This spectral evidence supports the physical and mechanical enhancements observed in prior sections of the study.

## Conclusion

This study comprehensively evaluated the multi-performance optimization of hybrid banana/bark cloth fiber-reinforced epoxy composites using the GRA technique, targeting application in automotive interior components. The integration of these natural fibers was aimed at achieving a balance between mechanical strength, flame retardancy, and water resistance, a multifaceted requirement critical to sustainable material development for transportation sectors. Through meticulous material selection, compression-moulded fabrication, and systematic testing (tensile, flexural, specific impact energy, flammability, and water absorption), the research established that hybridizing banana and bark cloth fibers significantly enhances performance attributes compared to single-fiber composites.

The optimal composite (Sample C7) demonstrated superior tensile strength (28.284 MPa), flexural strength (37.858 MPa), impact resistance (106.921 J/g), and the horizontal burning rate (1.053 mm/s), signifying a well-balanced structure suitable for automotive interior environments where safety and durability are paramount. The Grey Relational Analysis (GRA) ranked this configuration highest among all samples, validating GRA’s efficacy in multi-response optimization scenarios. Morphological analysis via SEM confirmed strong interfacial bonding and reduced fiber pull-out in the optimal sample, while EDAX and FTIR analyses substantiated the elemental and chemical compatibility of the fiber-matrix system.

The mechanical performance enhancement was primarily attributed to the synergistic effect of banana fiber’s tensile robustness and bark cloth’s laminar reinforcement, coupled with effective NaOH treatment and epoxy-ATH integration. The hybrid composite’s fire-retardant performance, particularly under ASTM D635 HB conditions, was strengthened by the inclusion of ATH (5 wt%) and the inherent thermal resistance of bark cloth. Moreover, the water absorption study over 10 days revealed that Sample C7 exhibits moderate saturation behavior, indicating improved fibre-matrix adhesion and moisture resistance. However, since void content was not quantitatively measured, any inference regarding porosity should be regarded as qualitative.

From a material-cost perspective, the hybrid C7 composite offers a substantial economic advantage. Banana and bark cloth fibers are considerably less expensive than conventional E-glass fibers, potentially reducing raw material costs by 40–70%. While the mechanical strength of the hybrid composite is comparatively lower, its favorable specific strength, enhanced flame retardancy, and low density make it well-suited for cost-sensitive and sustainable automotive interior applications.

Despite the promising findings, certain limitations were identified. The study was confined to epoxy as the matrix material; thus, a comparative analysis with other resins, such as PLA or polyester, remains unexplored. The experiments were conducted at a laboratory scale under controlled environmental conditions; therefore, scalability, long-term durability (e.g., UV exposure, biodegradation), and real-world automotive performance have yet to be assessed. Additionally, flame retardancy was evaluated using ATH only, leaving the influence of synergists and advanced coatings for future examination. Finally, while laminate consolidation quality was assessed through visual inspection and SEM features (e.g., minimal visible voids in the examined regions), a quantitative void fraction/porosity measurement was not performed; therefore, “void-free/minimal void” statements should be interpreted as qualitative rather than bulk-quantified laminate porosity. In addition, although all laminates were fabricated in a 3 mm mould cavity under the same compression load, differences in fibre density and packing between banana fibre and bark cloth mean that identical nominal thickness does not necessarily correspond to identical internal fibre volume fraction or porosity across all configurations.

### Future scope

Future studies will focus on evaluating the hybrid composites under accelerated aging, thermal cycling, and real-world automotive conditions to confirm long-term durability. Incorporating nano-fillers or bio-based synergists and integrating Machine Learning–assisted multi-objective optimization with GRA could further enhance performance prediction and material design. Comparative benchmarking with commercial composites and Life Cycle Assessments will be essential to establish the industrial viability of these sustainable materials.

## Supplementary Information

Below is the link to the electronic supplementary material.

Supplementary Material 1

## Data Availability

The data supporting the findings of this study are available as supplementary information.
