# Supplementary material for: Investigation of multi-performance optimization of banana/bark cloth reinforced epoxy composites using grey relational analysis for automotive interior applications
Source: Sci Rep. 2026 Mar 23;16:14615. doi: 10.1038/s41598-026-45783-9 (PMC13153230; doi:10.1038/s41598-026-45783-9)
Supplement: Supplementary file 1 — Supplementary Material 1 [file 41598_2026_45783_MOESM1_ESM.pdf]

Investigation of Multi-Performance Optimization of Banana/Bark Cloth Reinforced Epoxy Composites Using Grey Relational Analysis for Automotive Interior Applications

Alex Turyahabwe[1], [1]Department of Mechanical Engineering, School of Engineering and Applied Sciences, Kampala International University, Western Campus, Ishaka-Bushenyi, Uganda, https://orcid.org/0009-0006-6220-2961, Email: aleturyahabwe@gmail.com

Milon Selvam Dennison[2*], [2]Department of Mechanical Engineering, School of Engineering and Applied Sciences, Kampala International University, Western Campus, Ishaka-Bushenyi, Uganda, https://orcid.org/0000-0002-2012-9288, Email: milonds.mf@gmail.com

Onep Samuel George[3], [3]Department of Mechanical Engineering, School of Engineering and Applied Sciences, Kampala International University, Western Campus, Ishaka-Bushenyi, Uganda, https://orcid.org/0009-0009-6721-1981, Email: onep.samuel@kiu.ac.ug

*Corresponding Author: milonds.mf@gmail.com; milon.selvam@kiu.ac.ug

Supporting Information (SI)

SI 1. Flexural Strength

Rectangular specimens were set up on two support points, with a third loading point exerting force at the center. The span-to-thickness ratio was kept consistent to guarantee precise results, avoiding early failure from shear stress. The recorded force-deflection data was used to calculate the flexural modulus and strength. The maximum stress experienced by the specimen during the test, calculated using the **eqn. (1).**

$$\sigma_f = \frac{3PL}{2bd^2} \quad (1)$$

Where: σ_f = Flexural strength (MPa), P = Maximum load applied before fracture (N), L= Span length between the supports (mm), b= Width of the specimen (mm), d= Thickness of the specimen (mm). Flexural modulus is calculated from the slope of the initial linear region of the load-deflection curve and is given by **eqn. (2).**

$$E_f = \frac{L^3 m}{4bd^3} \quad (2)$$

Where: E_f= Flexural modulus (MPa), L= Span length between the supports (mm), m= Slope of the initial linear portion of the load-deflection curve (N/mm), b= Width of the specimen (mm), d= Thickness of the specimen (mm).

SI 2. Grey Relational Analysis (GRA)

Grey Relation Analysis (GRA) is an effective multi-objective optimization method that helps assess complex systems with various performance criteria. When it comes to improving the production of banana fibre/barkcloth composites, GRA plays a crucial role in identifying the optimum fabrication parameters by examining how various mechanical properties, like tensile strength, flexural strength, specific impact energy and horizontal burning are interconnected. The methodical approach followed in this research is as follows;

Step 1: Construct the Decision Matrix

The first step involves compiling the experimental results into a decision matrix. Each row represents an experimental trial, while each column represents a measured performance response (e.g., tensile strength, flexural strength, specific impact energy and horizontal burning rate).

$$\mathbf{X}= \begin{bmatrix} x_{11} & x_{12} & \cdots & x_{1m} \\ x_{21} & x_{22} & \cdots & x_{2m} \\ \vdots & \vdots & \ddots & \vdots \\ x_{n1} & x_{n2} & \cdots & x_{nm} \end{bmatrix}$$

Where:

x_{ij} represents the value of the jth performance criterion in the ith experiment.

n is the number of experiments.

m is the number of performance metrics (e.g., tensile strength, flexural strength, specific impact energy and horizontal burning rate).

Step 2: Normalize the Decision Matrix

Normalization ensures that all responses are scaled within the range 0 to 1, eliminating the effects of different units and magnitudes. The normalization method depends on the type of response variable:

Higher-the-Better (HTB): For responses that should be maximized (e.g., tensile strength, flexural strength, specific impact energy), the normalization formula is given as **eqn. (3)**

$$\boldsymbol{X_{ij}^{*}} = \frac{\boldsymbol{x_{ij} - min\ i_j}}{\boldsymbol{max\ x_j - min\ x_j}} \quad \textbf{(3)}$$

Lower-the-Better (LTB): For responses that should be minimized (e.g., horizontal burning rate), the normalization formula is given as **eqn. (4)**

$$\boldsymbol{X_{ij}^{*}} = \frac{\boldsymbol{Max\ x_{ij} - x_{ij}}}{\boldsymbol{max\ x_j - min\ x_j}} \quad \textbf{(3)}$$

Where:

X^*_{ij} is the normalized value.

Max xj and min xj are the maximum and minimum values for each response j.

The normalized matrix will have all values between 0 and 1, making them comparable.

Step 3: Compute the Grey Relational Coefficient (GRC)

The Grey Relational Coefficient (GRC) quantifies the relationship between each experimental trial and the ideal (best-performing) trial. It is computed using the formula is given as **eqn. (5)**

$$\boldsymbol{\xi_{ij} = \frac{\Delta_{min} + \varsigma\Delta_{max}}{\Delta_{ij} + \varsigma\Delta_{max}}} \quad \textbf{(5)}$$

Where:

ξ_{ij} is the Grey Relational Coefficient for the jth response in the ith experiment.

Δ_{ij} is the absolute difference between the normalized value and the ideal value (best-performing case).

Δ_{min} and Δ_{max} are the minimum and maximum absolute differences across all experiments.

ζ is the distinguishing coefficient, usually set between 0.1 and 0.5 (commonly 0.5). The GRC values range between 0 and 1, where higher values indicate better performance.

Step 4: Calculate the Grey Relational Grade (GRG)

The Grey Relational Grade (GRG) aggregates the GRC values across all performance metrics to obtain a single score for each experiment. The GRG is calculated as given in **eqn. (6)**

$$\boldsymbol{\gamma_i = \frac{1}{m}\sum_{j=1}^{m} \xi_{ij}} \quad \textbf{(6)}$$

Where:

γ_i is the Grey Relational Grade for the i_{th} experiment.

m is the number of performance metrics.

ξ_{ij} is the Grey Relational Coefficient for each response.

The higher the GRG, the better the overall performance of that experiment.

Step 5: Rank the Experiments and Identify Optimal Parameters

The experimental trials are ranked based on their GRG values. The highest-ranked trial corresponds to the optimum fabrication parameters that maximize performance.

Banana Fiber / Bark Cloth Composite - Experimental Plan					Results											
					Tensile Strength (MPa)	Flexural Strength (MPa)					Flexural Modulus		Drop Weight Impact Test			Horizontal Burning (HB) Test (mm/s)
Composition No.	Banana Fiber (wt%)	Bark Cloth Fiber (wt%)	Epoxy + Hardener (wt%)	Fire Retardant (ATH) (wt%)		d (mm)	L (mm)	b (mm)	P (N)	Flexural Strength (MPa)	Slope (m) N/mm	Flexural Modulus, E (MPa)	Impact Energy (J)	Mass of composite(g)	Specific Impact Energy (J/g)	
**C1**	10	35	50	5	4.693	3.010	48.000	12.700	11.816	7.394	118.159	9432.495	5427.677	48.530	111.842	0.952
**C2**	15	30	50	5	10.550	3.070	48.000	12.700	26.246	15.787	262.457	19747.108	5366.462	57.810	92.829	0.952
**C3**	20	25	50	5	17.876	3.100	48.000	12.700	50.105	29.559	501.055	36615.088	4754.318	58.960	80.636	0.794
**C4**	25	20	50	5	20.418	3.060	48.000	12.700	53.112	32.157	531.124	40354.485	4285.008	52.870	81.048	1.111
**C5**	30	15	50	5	20.904	3.050	48.000	12.700	59.420	36.213	594.197	45592.255	5121.605	51.990	98.511	0.870
**C6**	35	10	50	5	17.889	3.030	48.000	12.700	54.741	33.803	547.411	42839.635	5672.534	55.670	101.896	1.000
**C7**	40	5	50	5	28.284	3.020	48.000	12.700	60.904	37.858	609.040	48137.665	5346.058	50.000	106.921	1.053
**C8**	45	0	50	5	23.295	3.090	48.000	12.700	49.425	29.346	494.247	36469.383	4203.389	52.220	80.494	0.833
					**Larger the better**					**Larger the better**					**Larger the better**	**Smaller the better**
				Min	4.693					7.394					80.494	0.794
				Max	28.284					37.858					111.842	1.111

Step - 1 (Normalized Data)

Sample	TS	FS	SIE	HB
**C1**	0.000	0.000	1.000	0.500
**C2**	0.248	0.276	0.394	0.500
**C3**	0.559	0.728	0.005	1.000
**C4**	0.667	0.813	0.018	0.000
**C5**	0.687	0.946	0.575	0.761
**C6**	0.559	0.867	0.683	0.350
**C7**	1.000	1.000	0.843	0.184
**C8**	0.789	0.721	0.000	0.875
Min	0.000	0.000	0.000	0.000
Max	1.000	1.000	1.000	1.000

Step - 2 (Deviation Sequence)

Sample	TS	FS	SIE	HB
**C1**	1.000	1.000	0.000	0.500
**C2**	0.752	0.724	0.606	0.500
**C3**	0.441	0.272	0.995	0.000
**C4**	0.333	0.187	0.982	1.000
**C5**	0.313	0.054	0.425	0.239
**C6**	0.441	0.133	0.317	0.650
**C7**	0.000	0.000	0.157	0.816
**C8**	0.211	0.279	1.000	0.125
Min	0.000	0.000	0.000	0.000
Max	1.000	1.000	1.000	1.000

Step - 3 (Grey Relational Coefficient)

Sample	TS	FS	SIE	HB
**C1**	0.333	0.333	1.000	0.500
**C2**	0.399	0.408	0.452	0.500
**C3**	0.531	0.647	0.334	1.000
**C4**	0.600	0.728	0.337	0.333
**C5**	0.615	0.902	0.540	0.676
**C6**	0.532	0.790	0.612	0.435
**C7**	1.000	1.000	0.761	0.380
**C8**	0.703	0.642	0.333	0.800

Step - 4 (Grey Relational Grade)

Sample	TS	FS	SIE	HB	GRG
**C1**	0.333	0.333	1.000	0.500	0.542
**C2**	0.399	0.408	0.452	0.500	0.440
**C3**	0.531	0.647	0.334	1.000	0.628
**C4**	0.600	0.728	0.337	0.333	0.500
**C5**	0.615	0.902	0.540	0.676	0.684
**C6**	0.532	0.790	0.612	0.435	0.592
**C7**	1.000	1.000	0.761	0.380	0.785
**C8**	0.703	0.642	0.333	0.800	0.619

Step - 5 (Rank)

Sample	Rank
**C1**	6
**C2**	8
**C3**	3
**C4**	7
**C5**	2
**C6**	5
**C7**	1
**C8**	4
